# Safety and efficacy of clinical-grade, cryopreserved menstrual blood mesenchymal stromal cells in experimental acute respiratory distress syndrome

**DOI:** 10.3389/fcell.2023.1031331

**Published:** 2023-01-30

**Authors:** Francisca Alcayaga-Miranda, Johnatas Dutra Silva, Nicol Parada, Luisa Helena Andrade da Silva, Fernanda Ferreira Cruz, Yildy Utreras, Yessia Hidalgo, María Ignacia Cádiz, Rafael Tapia Limonchi, Francisco Espinoza, Alejandro Bruhn, Maroun Khoury, Patricia R. M. Rocco, Jimena Cuenca

**Affiliations:** ^1^ Laboratory of Nano-Regenerative Medicine, Centro de Investigación e Innovación Biomédica (CIIB), Faculty of Medicine, Universidad de los Andes, Santiago, Chile; ^2^ Consorcio Regenero, Chilean Consortium for Regenerative Medicine, Santiago, Chile; ^3^ Cells for Cells, Santiago, Chile; ^4^ IMPACT, Center of Interventional Medicine for Precision and Advanced Cellular Therapy, Santiago, Chile; ^5^ Laboratory of Pulmonary Investigation, Carlos Chagas Filho Biophysics Institute, Federal University of Rio de Janeiro, Rio de Janeiro, Brazil; ^6^ National Institute of Science and Technology for Regenerative Medicine, Rio de Janeiro, Brazil; ^7^ Departamento de Medicina Intensiva, Facultad de Medicina, Pontificia Universidad Católica de Chile, Santiago, Chile

**Keywords:** fresh MenSCs, ARDS (acute respiratory disease syndrome), MenSCs (menstrual blood-derived mesenchymal stromal cells), cryopreserved MenSCs, off-the-shelf MenSCs

## Abstract

**Background:** Treatment for critical care conditions, such as acute respiratory distress syndrome (ARDS), requires ready-to-administer injectable mesenchymal stromal cells (MSCs). A validated cryopreserved therapy based on MSCs derived from menstrual blood (MenSCs) is an attractive option that offers advantages over freshly cultured cells and allows its use as an off-the-shelf therapy in acute clinical conditions. The main goal of this study is to provide evidence on the impact of cryopreservation on different biological functions of MenSCs and to determine the optimal therapeutic dose, safety, and efficacy profile of clinical-grade, cryopreserved (cryo)-MenSCs in experimental ARDS.

**Methods:** Biological functions of fresh versus cryo-MenSCs were compared *in vitro*. The effects of cryo-MenSCs therapy were evaluated *in vivo* in ARDS-induced (*Escherichia coli* lipopolysaccharide) C57BL/6 mice. After 24 h, the animals were treated with five doses ranging from 0.25×10^5^ to 1.25×10^6^ cells/animal. At 2 and 7 days after induction of ARDS, safety and efficacy were evaluated.

**Results:** Clinical-grade cryo-MenSCs injections improved lung mechanics and reduced alveolar collapse, tissue cellularity, and remodelling, decreasing elastic and collagen fiber content in alveolar septa. In addition, administration of these cells modulated inflammatory mediators and promoted pro-angiogenic and anti-apoptotic effects in lung-injured animals. More beneficial effects were observed with an optimal dose of 4×10^6^ cells/Kg than with higher or lower doses.

**Conclusion:** From a translational perspective, the results showed that clinical-grade cryopreserved MenSCs retain their biological properties and exert a therapeutic effect in mild to moderate experimental ARDS. The optimal therapeutic dose was well-tolerated, safe, and effective, favouring improved lung function. These findings support the potential value of an off-the-shelf MenSCs-based product as a promising therapeutic strategy for treating ARDS.

## Introduction

Acute respiratory distress syndrome (ARDS) is the most severe form of respiratory failure and accounts >10% of intensive care unit (ICU) admissions worldwide (Laffey and Matthay, 2017). Unfortunately, this number has increased as the world faces the COVID-19 pandemic, with up to 31% of patients developing ARDS (Li and Ma, 2020). According to recent studies, the mortality rate is higher than 40% for typical ARDS (Laffey and Matthay, 2017) but may range between 26% and 66% for COVID-19 ARDS (Gibson et al., 2020). There is still no specific therapy for ARDS besides supportive care (Bellani et al., 2016).

Mesenchymal stromal cells (MSCs)-based therapies have attracted considerable interest as a potential treatment for ARDS due to their ability to decrease lung inflammation and improve respiratory function (Laffey and Matthay, 2017). It is worth mentioning that clinical and pilot uncontrolled trials based on MSCs experienced an explosive increase due to the global pandemic of COVID-19 (Khoury et al., 2020). The systematic reviews and meta-analyses examining clinical studies based on the use of MSCs for COVID-19 ARDS agree that MSCs were safe, showed no detection of serious adverse events and were associated with a reduction in mortality and time to recovery compared to controls (Masterson et al., 2021; Chen et al., 2022; Kirkham et al., 2022).

Administration of diverse MSCs sources are known to display distinct functional properties in respiratory diseases (Antunes MA, Abreu SC Antunes et al., 2014; Silva JD, Lopes-Pacheco Silva et al., 2018) and other pathologies (Akimoto et al., 2013; Sugiyama et al., 2018), which might present specific therapeutic effects. However, the best source of MSCs for ARDS remains undetermined. Menstrual blood-derived mesenchymal stromal cells (MenSCs) have several advantages, such as no ethical concerns, non-invasive acquisition, easy isolation, multiple collections, low immunogenicity, high proliferative rate, and potent angiogenic and immunomodulatory properties shown both *in-vitro* (Meng et al., 2007; Alcayaga-Miranda et al., 2015a) and *in-vivo* (Alcayaga-Miranda et al., 2015b; Cuenca et al., 2018). MenSCs have been assessed in several disease animal models, including myocardial infarction (Hida et al., 2008), pulmonary fibrosis (Chen et al., 2020), and diabetes mellitus (Wu et al., 2014), among others (Chen et al., 2017; Xiang et al., 2017). Our group also demonstrated that treatment with MenSCs promoted skin wound repair (Cuenca et al., 2018), showed increased survival in graft versus host disease (Luz-Crawford et al., 2016), and prevented acute lung injury in experimental sepsis (Alcayaga-Miranda et al., 2015b).

The development of MSCs-based therapy faces several challenges: to establish the optimal dosage of cells at the correct timing to obtain maximum therapeutic benefit in the clinical setting (Giri and Galipeau, 2020), and to transfer these therapies from bench to the ICU without compromising product potency and quality because ARDS is an acute condition that requires immediate intervention. Therefore, a cryopreserved allogeneic MenSCs product may be an attractive option because it offers a longer shelf-life and stability, ability to store large amounts, and production of an immediate, off-the-shelf therapeutic supply for rapid administration (Luetzkendorf et al., 2015; Woods et al., 2016). Although off-the-shelf cryopreserved MSCs products have been used in several clinical trials, including ARDS (Wilson et al., 2015), controversies remain regarding their potency or efficacy compared with fresh MSCs (François et al., 2012; Cruz et al., 2015; Gramlich et al., 2016).

The present study evaluated the safety and efficacy of an off-the-shelf treatment with clinical-grade cryopreserved MenSCs at different doses and time points in experimental ARDS. We also compared different biological functions of fresh versus cryopreserved MenSCs *in-vitro*.

## Material and methods

Additional details are available in the online Supplementary Material.

### MSCs culture, cryopreservation, and final preparation

Clinical-grade cryo-MenSCs were isolated and characterized as described previously (Alcayaga-Miranda et al., 2015a) following good manufacturing practice. Briefly, the cells were cryopreserved at a concentration of 1.25×10^6^ cells/mL in fetal bovine serum (FBS) 90% (Gibco, Waltham, MA, United States) and dimethyl sulfoxide (DMSO) 10% (Sigma, St. Louis, MO, United States) with controlled-rate freezing (CryoMed, Thermo Fisher Scientific, Waltham, MA, United States) and stored in liquid nitrogen (2–4 weeks) until use. Cells cultured for at least 7 days before *in-vitro* assays are called fresh MenSCs, and MenSCs thawed and washed immediately before testing are called cryo-MenSCs. All MenSCs tested negative for *mycoplasma* (PCR *Mycoplasma* Detection Kit, abm, Richmond, BC, Canada), endotoxin (≤0.05 EU/mL; Endosafe, Charles River, Wilmington, MA, United States), and microbiological growth (Laboratory of Microbiology, Clínica Universidad de los Andes, Las Condes, Chile). These cryo-MenSCs were compared with fresh MenSCs, evaluating their cell surface markers, viability, senescence, karyotyping, adhesion potential, proliferative activity, differentiation abilities, colony-forming unit (CFU-F), and immunosuppressive capacity (Supplementary Figure S1 summarizes the experimental studies).

### Mouse model of ARDS and MenSCs treatment protocol

**FIGURE 1:**
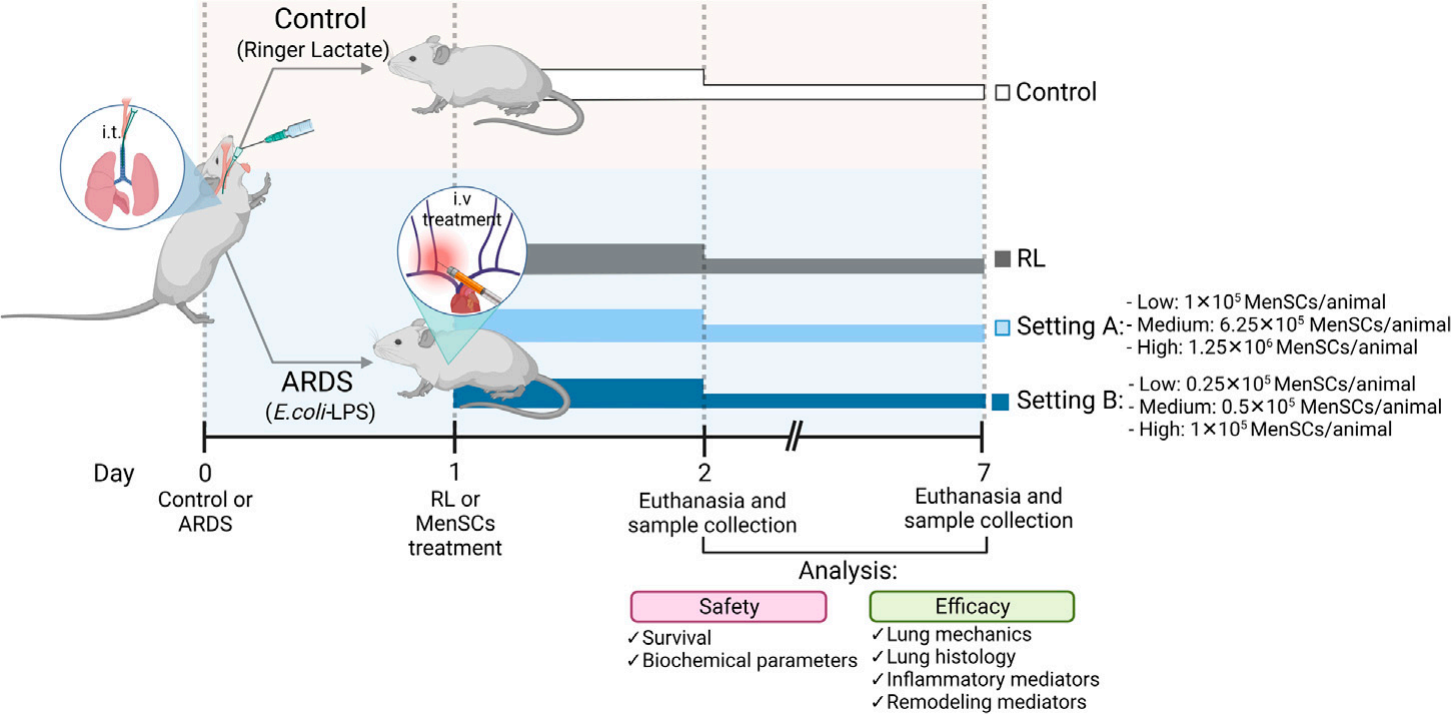
Scheme of the *in vivo* protocol for ARDS induced by LPS and treatment with cryopreserved-MenSCs. Flowchart of the experimental procedures for the ARDS mouse model. Animals were randomly assigned to be in the control (CTRL) group or the ARDS group. The CTRL group received 50 μL of Ringer’s lactate (RL), and the ARDS group received 50 μL of *Escherichia coli* lipopolysaccharide (LPS) (2 mg/kg), intratracheally (day 0). After 24 h (day 1), the animals with ARDS were randomly divided into those that were treated intravenously (50 μL) through the jugular vein with RL (ARDS-RL group) or with MenSCs (ARDS-MenSCs group) at different doses according to each setting, A or B (n = 8–10 animals/group; setting A: 1×10^5^, 6.25×10^5^ and 1.25×10^6^ cryo-MenSCs/mouse; setting B: 0.25×10^5^, 0.5×10^5^ and 1×10^5^ MenSCs/mouse). The evaluation and euthanasia were on days 2 and 7 after ARDS induction or RL administration. Figure created with BioRender.com.

The experimental animal model was performed as described previously (Maron-Gutierrez et al., 2013; Silva et al., 2014). Figure 1 summarizes the study design. Briefly, C57BL/6 mice (male, 20–25 g, 8–10 weeks) were administered intratracheally with Ringer’s lactate (RL) (50 μL, control group [CTRL]) or *Escherichia coli* lipopolysaccharide (LPS; Ultrapure; InvivoGen, San Diego, CA, United States); 50 μL, 2 mg/kg, ARDS group). After 24 h, the animals injected with LPS were randomized into subgroups receiving RL (50 μL, ARDS-RL group) or cryo-MenSCs intravenously (IV) in the internal jugular vein (different doses in 50 μL RL/mouse, ARDS-MenSCs group). The cryo-MenSCs doses (0.25×10^5^, 0.5×10^5^, 1×10^5^, 6.25×10^5^ and 1.25×10^6^ cryo-MenSCs/mouse) used in each experimental setting, A (higher doses) or B (lower doses), are indicated in Figure 1. The lung analysis was performed at 2 and 7 days after induction of ARDS, and then the animals were euthanized.

### Analysis of lung mechanics

At 2 and 7 days after LPS or RL administration, animals were sedated, anaesthetized, tracheotomized, paralyzed, and mechanically ventilated as described previously (Leite-Junior et al., 2008). After chest resection, wall static lung elastance (Est), resistive (ΔP1), and viscoelastic pressures (ΔP2) were obtained using the end-inflation occlusion method (Bates et al., 1985). ΔP1 selectively reflects the pressure used to overcome the airway resistance. ΔP2 reproduces the pressure spent by stress relaxation, or the viscoelastic properties of the lung, together with a small contribution of pendelluft. All data were analysed using the ANADAT software package (RHT-InfoData) as previously (Leite-Junior et al., 2008).

### Histological analysis

After perfusion and fixation in 4% buffered formaldehyde solution (Sigma-Aldrich), lung tissue samples were embedded in paraffin using standard protocols, cut into 4-μm-thick slices, and stained with haematoxylin-eosin (HE) (Sigma-Aldrich, St. Louis, MO, United States). Diffuse alveolar damage (DAD) was semi-quantified in a blinded manner using a weighted scoring system as described previously (Oliveira et al., 2015). In brief, score values from 0 to 4 represent the severity of alveolar collapse, interstitial oedema, inflammation, and septal thickening (0, no effect; 4, maximum severity). In addition, the extent of each scored feature per field of view was determined on a scale of 0–4 (0, no visible evidence; 4, complete involvement). Scores were calculated as the product of severity and extent of each feature, ranging from 0 to 16. The cumulative DAD score was calculated as the sum of the product of the severity of the four features (0–64). Specific staining methods were used to quantify collagen (Picrosirius polarization method) and elastic fibers (Weigert’s resorcin-fuchsin method modified with oxidation) in the alveolar septa (Leite-Junior et al., 2008). Van Gieson staining was also performed to analyze the wall thickness and adventitia for the small pulmonary arteries as described previously (Castillo-Galán et al., 2020).

Immunohistochemistry was performed to detect caspase-3 (rabbit polyclonal 1:1000; Cell Signaling Technology, Beverly, MA, United States), CD31 (rabbit polyclonal 1:50; Abcam, Cambridge, MA, United States), and Ku80 (rabbit monoclonal 1:800; Abcam) using the Dako Envision system (Dako, Carpinteria, CA, United States) according to the manufacturer’s recommendations. Immunoreactive sections were visualized with diaminobenzidine (DAB) solution (Dako) and counterstained with haematoxylin. Semiquantitative histological scoring was performed in a blinded fashion ranging from 0 (non-specific stain) to 4 (high specific stain).

### Determination of inflammatory mediators

Levels of keratinocytes-derived chemokine (KC/CXCL1), interleukin (IL)-1β, IL-6, tumour necrosis factor alpha (TNFα), and C-C motif chemokine ligand 2 (CCL2) were determined in lung tissue homogenates using commercial ELISA kits (R&D Systems, Minneapolis, MN, United States) according to the manufacturer’s instructions. Expression levels of inducible nitric oxide synthase (iNOS) and arginase 1 (Arg-1) were determined with total RNA extracted using a RNeasy kit (QIAGEN, Marseille, France) from harvested lung tissue and qPCR was performed at Stratagene Mx3000P (Agilent Technologies, Santa Clara, CA, United States). All values were normalized to GAPDH as housekeeping gene and expressed as fold change or relative expression using the 2^−ΔΔCT^ formula (Livak and Schmittgen, 2001).

### Blood biochemical analysis

Plasma levels of creatinine, blood urea nitrogen, alanine aminotransferase, aspartate aminotransferase, alkaline phosphatase, albumin, amylase, and total protein were evaluated as previously (Alcayaga-Miranda et al., 2015b) by Piccolo General Chemistry 13 using the Piccolo Xpress Chemistry Analyzer (Abaxis, Union City, CA, United States) following the manufacturer’s instructions.

### Statistical analysis

Data are expressed as means ± standard error of mean (SEM) unless otherwise specified. The distribution of all data was tested for normality. Unpaired, two-tailed, Student’s t test was performed to compare two different groups. ANOVA followed by Dunnett’s post-test was used for the analysis of multiple comparison groups. Non-parametric data were analysed using ANOVA on ranks followed by Kruskal–Wallis or Dunn’s *post hoc* test. The number of samples (n) per group is specified in the figure legends. All tests were performed using the GraphPad Prism 9 statistical software package (GraphPad Software, La Jolla, CA, United States). A *p*-value ≤ 0.05, was considered statistically significant.

## Results

### Cryo-MenSCs and fresh MenSCs display similar properties *in vitro*

**FIGURE 2:**
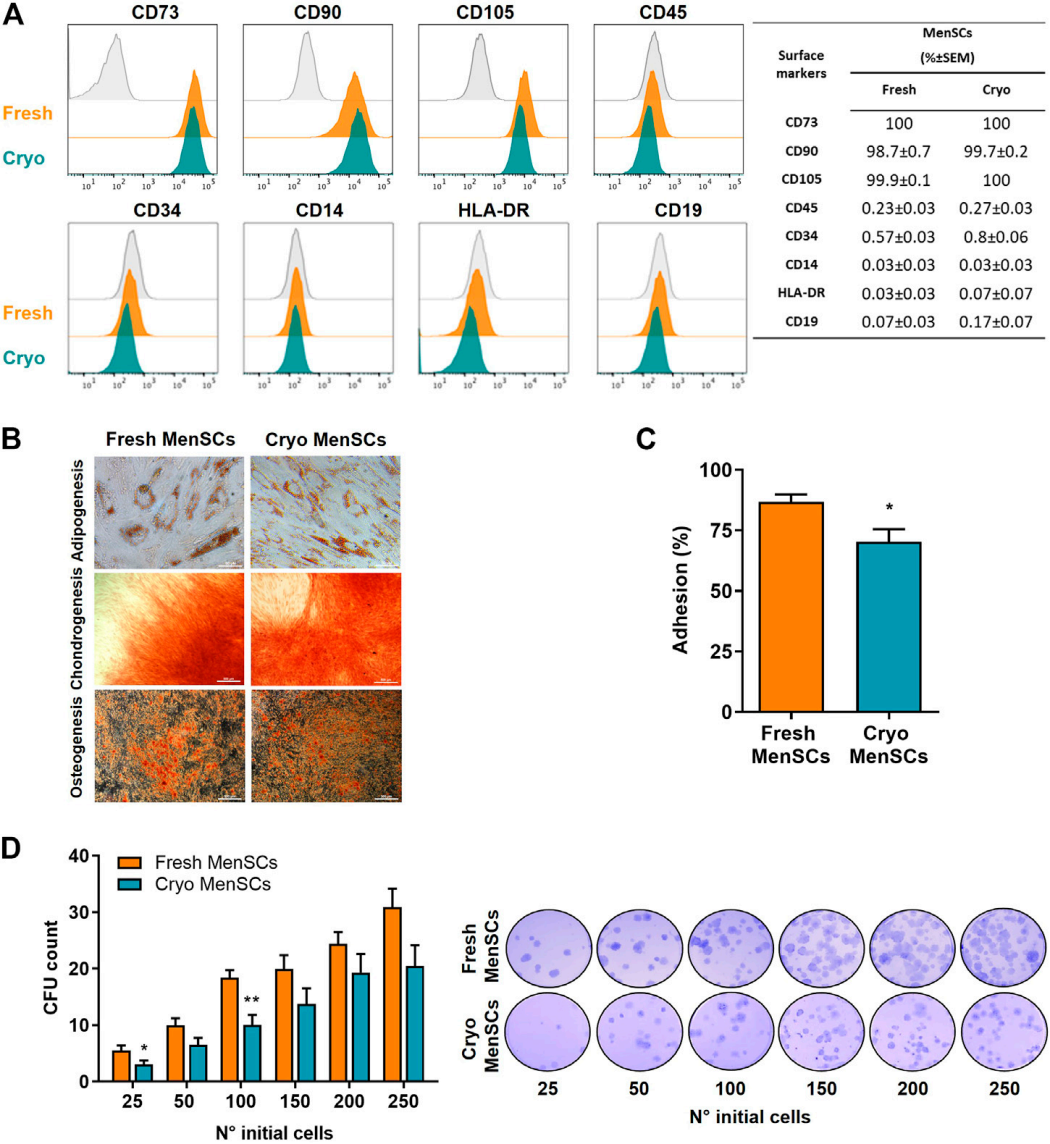
Comparison studies of cryopreserved and fresh MenSCs: immunophenotype, differentiation capacity, colony-forming unit potential, and adhesion. The analyses were performed with cryopreserved and thawed cells (cryo-MenSCs) compared with fresh culture cells (fresh MenSCs). **(A)** Flow cytometric analysis of MenSCs surface markers. Representative histograms of positive (CD73, CD90, and CD105) and negative (CD45, CD34, CD14, HLA-DR, and CD19) markers (orange and green lines) and the autofluorescence control from fresh MenSCs (grey line). Viable cells were determined using a vital dye (LIVE/DEAD). The quantity of each marker is shown in the table. **(B)** MenSCs displayed mesodermal tri-differentiation capacity. Representative images of MenSCs differentiation after specific induction media and staining for adipocytes (Oil red O), chondrocytes (Safranin O), and osteocytes (Alizarin Red). **(C)** Adhesion to the plastic or cell culture surface of MenSCs after 24 h in culture. **(D)** Quantification of colony-forming units (CFU) of MenSCs. Cells were seeded at the indicated number in complete medium and cultured for 14 days. Representative images of fixed and stained cultures are shown on the right. Values are expressed as the mean ± standard error of the mean (SEM) of three different MenSCs donors and at least three independent experiments; ^*^ *p* ≤ 0.05 and ^**^ *p* ≤ 0.01 (Cryo vs. Fresh).

Immunophenotypic analysis indicated similar surface marker profiling for both cryo-MenSCs and fresh MenSCs (identity ≥95%, CD73, CD90, and CD105 and purity ≤2%, CD14, CD19, CD34, CD45, and HLA-DR) (Figure 2A). In addition, both types of MenSCs exhibited potential for tri-differentiation, as confirmed by histological staining (Figure 2B, Supplementary Figure S2). Adhesion ability was higher in fresh MenSCs than cryo-MenSCs (86.8% ± 3.1% versus 70.3% ± 5.2%, respectively; *p* = 0.0176) (Figure 2C), and a colony-forming unit-fibroblast (CFU-F) assay showed that cryo-MenSCs exhibited marginally impaired CFU-F potential (Figure 2D).

**FIGURE 3:**
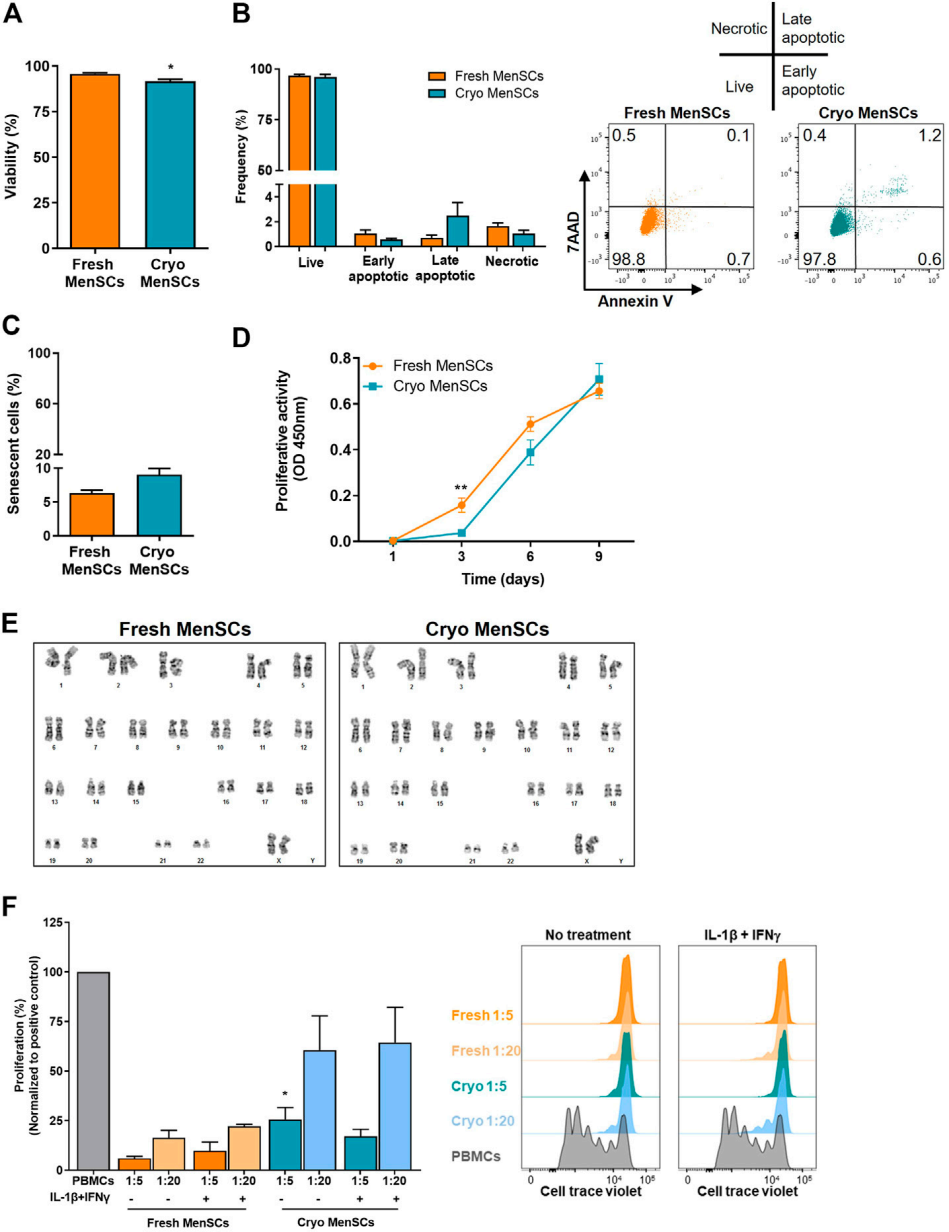
Viability, proliferative activity, senescence, karyotyping, and immunosuppressive properties of cryopreserved and fresh MenSCs. The analyses were performed with cryopreserved and thawed cells (Cryo-MenSCs) compared with fresh culture cells (Fresh-MenSCs). **(A)** Cell viability was determined by trypan blue dye exclusion assay. **(B)** Bar graph of apoptosis and necrosis, quantified by flow cytometry, after Annexin V and 7-aminoactinomycin D (7AAD) labelling. Representative dot plots showing intact cells in the lower-left quadrant (Q4); early apoptotic cells in the lower-right quadrant (Q3); late apoptotic cells in the upper-right quadrant (Q2), and necrotic cells in the upper-left quadrant (Q1). **(C)** Senescence-associated β-galactosidase. MenSCs were cultured and harvested under standard conditions. Cells were viewed by phase contrast on a microscope and photographed with a digital camera. Positively stained cells for senescence-associated (SA)-β-galactosidase were counted, and the percentage of cells expressing SA-β-galactosidase in the total cell number of each sample was calculated. **(D)** Metabolic activity was measured at days 1, 3, 6, and 9 after seeding using WST-1 reagent (optical density [OD], 450 nm). **(E)** Karyotype of MenSCs by GTG banding. Representative images of cryo- and fresh cells. To study the immunosuppressive capacity of MenSCs on T cell response, an *in vitro* T cell proliferation assay was performed at different ratios of MSCs/T cells, as described in the Material and Methods section. MenSCs were either unstimulated or were stimulated with interleukin (IL)-1β+interferon-γ (IFNγ) (10 ng/ml each) for 24 h before T cells co-culture in complete RPMI medium. Previously, human peripheral blood mononuclear cells (PBMCs) were labelled with Cell Trace Violet and stimulated with phytohaemagglutinin for 3–4 days. T cell proliferation was evaluated by flow cytometry, gating on CD45^+^CD3^+^ cells. **(F)** Graphical presentation of the quantified data and representative histograms of T cell proliferation in the presence and absence of MenSCs. Values are expressed as the mean ± standard error of the mean of three different MenSCs donors and at least three independent experiments. ^*^ *p* ≤ 0.05; ^**^ *p* ≤ 0.01 (Cryo versus Fresh).

Analysis of viability showed >90% viable cells by trypan blue exclusion assay (cryo-MenSCs, 91.6 ± 1.3; fresh MenSCs, 95.6 ± 0.6, *p* = 0.0139) (Figure 3A), which was confirmed by flow cytometry using 7-aminoactinomycin D (7ADD) where the viability was 95.7% ± 2% and 96.6% ± 0.4% for cryo-MenSCs and fresh MenSCs, respectively (Figure 3B). In a subsequent analysis of Annexin V (AV)/7ADD staining, showed no differences in the proportions of early/late apoptotic and necrotic cells (AV+/7ADD+) between cryo-MenSCs and fresh MenSCs (Figure 3B). The proliferation activity in cryo-MenSCs was lower than in fresh MenSCs at day 3 (0.04 ± 0.01 and 0.1 ± 0.03, *p* = 0.0045, respectively); however, the activity of cryo-MenSCs and fresh MenSCs increased to similar levels at days 6 and 9 (Figure 3D). To determine if cryopreservation could accelerate the induction of a senescent state, we evaluated the expression of β-galactosidase. No differences were observed (Figure 3C); a small percentage of cells were in a senescent state in both conditions (cryo-MenSCs, 9% ± 0.9%; fresh MenSCs, 6.3% ± 0.4%). The genetic stability of cryo-MenSCs, using karyotype analysis, revealed normal karyotype without numerical or structural aberrations after cryopreservation (Figure 3E).

To determine whether cryo-MenSCs maintain their immunomodulatory properties, MSCs were treated without and with IL-1β and interferon-γ (IFNγ) before co-culture with PBMCs. This *in-vitro* licensing best recapitulates what likely happens clinically once MSCs are transfused into patients with a dysregulated immune response (Galipeau et al., 2015). As shown in Figure 3F, cryo-MenSCs and fresh MenSCs displayed dose-dependent immunosuppressive properties, including cytokine-treated MSCs. Cryo-MenSCs exhibited a slightly lower potential to inhibit T cell proliferation than fresh cells. However, this difference was only significant (*p* = 0.0296) at a ratio 1:5 and independent of cytokine-treatment conditions (Figure 3F).

Overall, these data indicate that cryo-MenSCs versus fresh cells, exhibit marginally impaired biological characteristics.

### Effect of cryo-MenSCs treatment on animal survival

**FIGURE 4:**
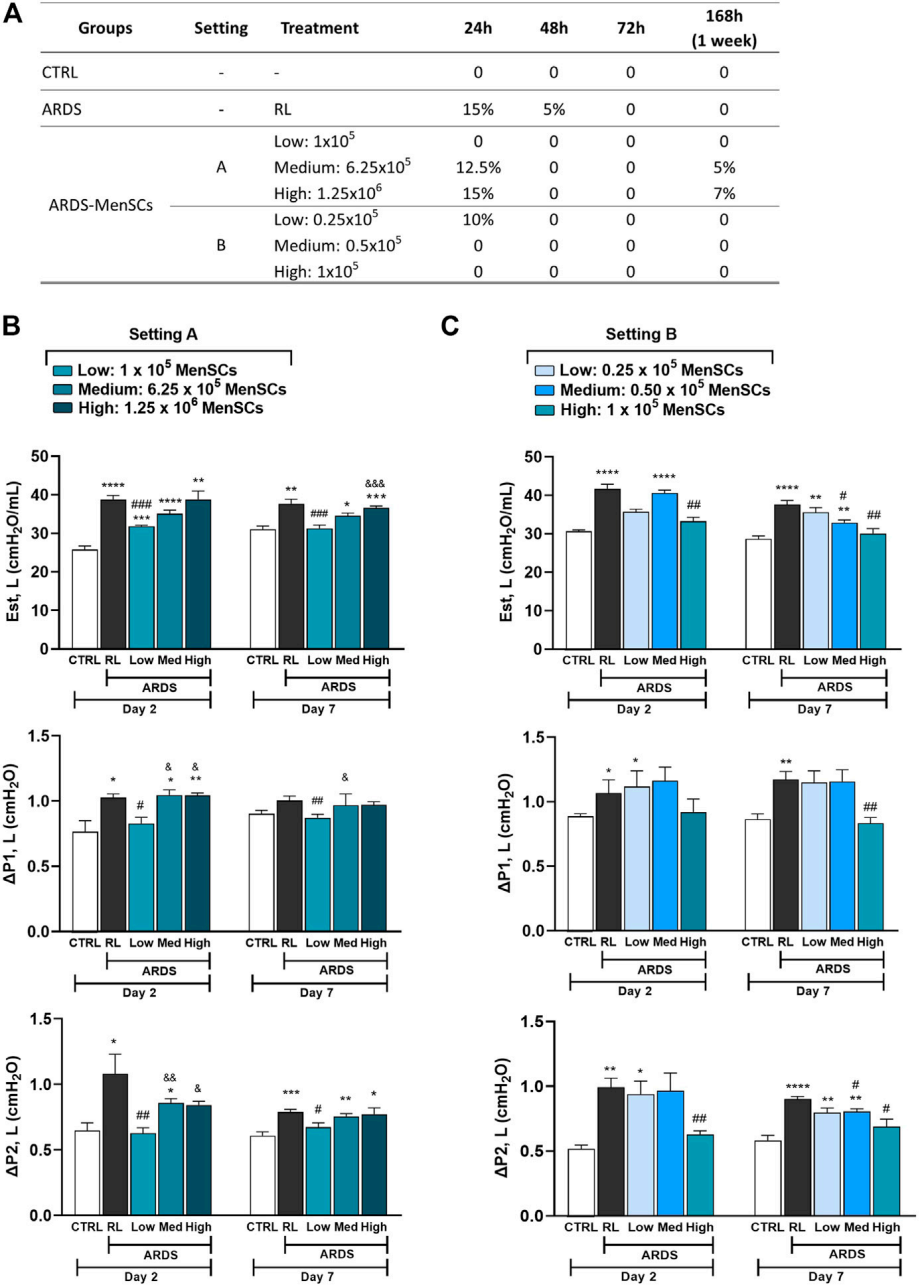
Treatment with cryopreserved MenSCs improves the mortality rate and lung mechanics after induction of ARDS. Animals were randomly assigned to the control (CTRL) group or the ARDS group and received Ringer’s lactate (RL) or *Escherichia coli* lipopolysaccharide (LPS), respectively. After 24 h (day 1), the animals with ARDS were randomly divided into those that were treated intravenously with RL (ARDS RL group) or with cryo-MenSCs (ARDS MenSCs group) at different doses according to each setting, A or B (n = 8–10 animals/group). **(A)** Mortality rate for the ARDS mouse model with cryo-MenSCs at different treatment regimes. **(B**, **C)** Effect of MenSCs on lung mechanics. The evaluation of static lung elastance (Est, L), resistive (ΔP1), and viscoelastic (ΔP2) pressures was performed on days 2 and 7 after LPS or RL administration at **(B)** setting A and **(C)** setting B. Values are expressed as the mean ± standard error of the mean. *Significantly different from the CTRL group (^*^ *p* ≤ 0.05, ^**^ *p* ≤ 0.01, ^***^ *p* ≤ 0.001;^****^ *p* ≤ 0.0001). **#**Different from the RL group (^#^ *p* ≤ 0.05, ^##^ *p* ≤ 0.01, ^###^ *p* ≤ 0.001). ^&^Different from the low MenSCs dose (^&^ *p* ≤ 0.05, ^&&^ *p* ≤ 0.01, ^&&&^ *p* ≤ 0.001).

In MSCs-based therapies, careful dose monitoring is required. Two experimental settings (Figure 1) were developed with different doses (low, medium, and high) to determine the minimum effective dose. Cryo-MenSCs at different doses (setting A: 1×10^5^, 6.25×10^5^; 1,25×10^6^; setting B: 0.25×10^5^; 0.5×10^5^; 1×10^5^ cells/animal) were injected IV 24 h after LPS-induced ARDS. The mortality rate in setting A at the lower dose of cryo-MenSCs (1×10^5^) was 0% compared with the untreated group (ARDS-RL) (Figure 4A). Unexpectedly, the medium (6.25×10^5^) and higher doses (1.25×10^6^) of cryo-MenSCs led to 12.5% and 15% mortality, respectively, similar to those observed in the ARDS-RL group (15%). In contrast, in setting B, the lower dose of 0.25×10^5^ cryo-MenSCs/animal only partially reduced mortality (10%) compared with the ARDS-RL group (15%); however, both the medium (0.5×10^5^) and higher (1×10^5^) doses showed 100% survival, confirming the previous result of setting A for the 1×10^5^ cryo-MenSCs dose (Figure 4A). These results suggest that high doses may not present advantages and that a minimum dose would also be ineffective in reducing mortality.

### Cryo-MenSCs treatment improved lung mechanics

In the analysis of lung mechanics, parameters for overcoming airway resistance (△P1, L), stress relaxation or viscoelastic properties (△P2, L), and lung static elastance (Est, L) were determined (Figure 4B). As expected, the ARDS-RL group showed higher Est, △P1, and △P2 compared with the CTRL group. Notably, of the five doses studied in both settings (A and B), only treatment with the 1×10^5^ cryo-MenSCs dose effectively reduced the lung mechanics parameters at the two-time points studied compared with the ARDS-RL group. In contrast, higher cryo-MenSCs doses such as 6.25×10^5^ and 1.25×10^6^ (setting A) or lower doses such as 0.25×10^5^ and 0.5×10^5^ (setting B) did not confer any improvement in these mechanics variables (Figures 4B, C).

### Cryo-MenSCs reduced lung morphologic changes and remodeling process

**FIGURE 5:**
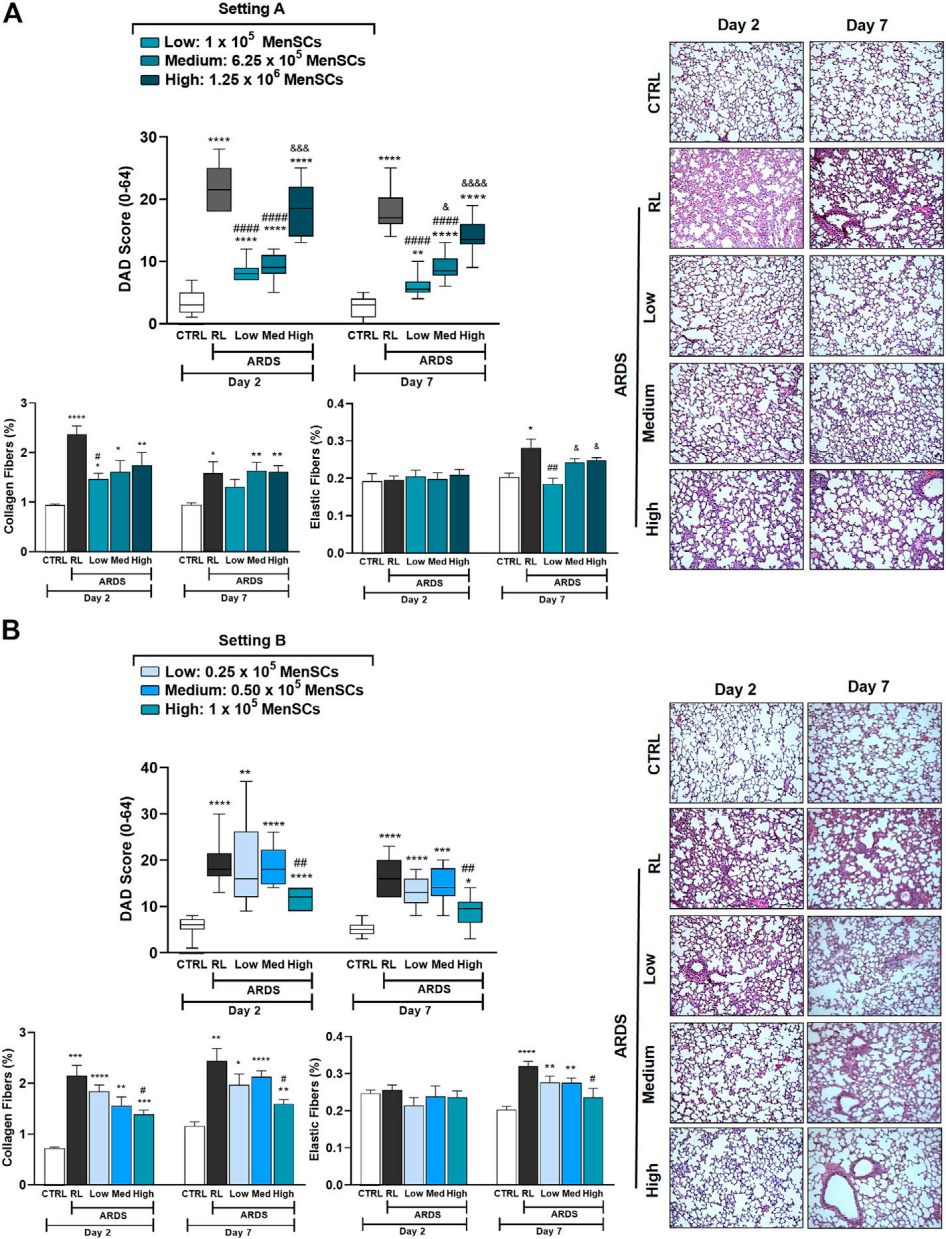
Reduction of the diffuse alveolar damage (DAD) score and lung tissue remodeling in lung-injured animals. The cumulative DAD score was calculated at **(A)** setting A and **(B)** setting B as the sum of each characteristic score (alveolar collapse, inflammatory infiltration, interstitial oedema, and septal thickening), and ranged from 0 to 64. Right: DAD score plots, representative images of lung parenchyma stained with haematoxylin-eosin. Magnification, ×100. Scale bars, 50 μm. Values are expressed as medians, interquartile ranges. For **(A)** setting A and **(B)** setting B, lung tissue samples were treated, and specific staining methods were used to quantify the content (%) of collagen fibers (Picrosirius polarization) and elastic fibers (Weigert’s resorcin-fuchsin, modified with oxidation), at days 2 and 7 after LPS or RL administration. Values are expressed as the mean ± standard error of the mean. ^*^Significantly different from the CTRL group (^*^ *p* ≤ 0.05, ^**^ *p* ≤ 0.01, ^***^ *p* ≤ 0.001; ^****^ *p* ≤ 0.0001). ^#^Different from the RL group (^#^ *p* ≤ 0.05, ^##^ *p* ≤ 0.01, ^####^ *p* ≤ 0.0001). ^&^Different from the low MenSCs dose (^&^ *p* ≤ 0.05, ^&&&^ *p* ≤ 0.001, ^&&&&^ *p* ≤ 0.0001).

To examine the effects of cryo-MenSCs treatments on lung damage and the remodeling process associated with ARDS, the diffuse alveolar damage (DAD) score, the fibers content (collagen and elastic), α-SMA and wall thickness and adventitia of the small pulmonary arteries were evaluated (Figures 5A, B; Supplementary Figure S3). As observed for lung mechanics (Figures 4B, C), the ARDS-RL group exhibited a significant increase in all four parameters studied compared with the CTRL group (Supplementary Figure S4). On days 2 and 7, the groups treated with 1×10^5^ and 6.25×10^5^ cryo-MenSCs, the severity of DAD score parameters, such as alveolar collapse, interstitial oedema, inflammation, and septal thickening, was reduced compared with the ARDS-RL group (Figure 4). Groups treated with the other doses studied (0.25×10^5^, 0.5×10^5^, or the highest dose of 1.25×10^6^ cryo-MenSCs/animal) had scores similar to the ARDS-RL group (Figure 5).

ARDS induction in animals promotes an increase in lung remodeling, as shown in Figure 5, which shows a marked increase in collagen and elastic fibers in the alveolar septum in the ARDS-RL group versus the CTRL group. In agreement with the DAD score, treatment with 1×10^5^ cryo-MenSCs significantly decreased the collagen levels at both time points compared with the ARDS-RL group. The evaluation of the elastic fiber content on day 2 showed no changes in any of the groups studied; however, this parameter was decreased only in the group that received 1×10^5^ cryo-MenSCs compared with the ARDS-RL group on day 7 (Figure 5). Additionally, ARDS induced an increase in the α-SMA immunoreactivity compared to the CTRL group on days 2 and 7; however, the cryo-MenSCs-treatment with 1×10^5^ and 1.25 × 10^6^ doses reduced α-SMA on both time points studied. Consequently, ARDS-RL animals also demonstrated increased medial and adventitial layers of small pulmonary arteries compared to CTRL; however, after administration of cryo-MenSCs these parameters were reduced, particularly with the 1×10^5^ dose (Supplementary Figure S3).

The above results indicate that the minimum dose to improve survival, lung mechanics, and remodeling was 1×10^5^ cryo-MenSCs/animal. The following experiments were conducted using doses of 0.25×10^5^, 1×10^5^, and 1.25×10^6^ cryo-MenSCs/animal to study some of the mechanisms involved in the observed effects.

### Cryo-MenSCs administration modulated inflammatory mediators

**FIGURE 6:**
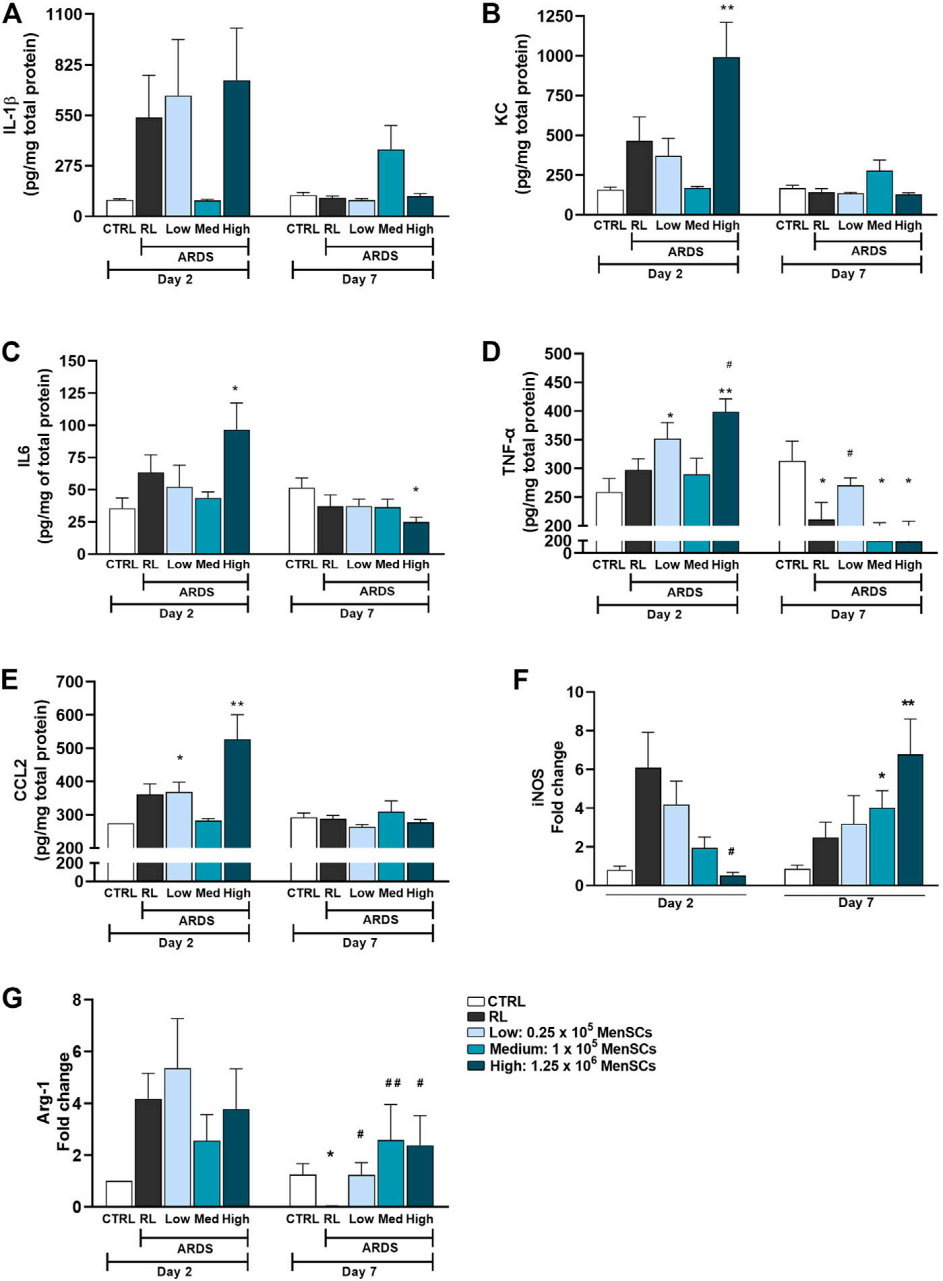
Cryopreserved MenSCs administration modulated inflammatory mediators. **(A**–**E)** Levels of inflammatory mediators determined by ELISA in lung tissue homogenates. Values are expressed as the mean ± standard error of the mean (SEM). **(F**, **G)** Expression of mRNA levels of iNOS and Arg-1. Data are expressed as fold change compared with the non-treated controls. Values are expressed as the mean ± SEM. ^*^Significantly different from the CTRL group (^*^ *p* ≤ 0.05, ^**^ *p* ≤ 0.01). ^#^Different from the RL group (^#^ *p* ≤ 0.05, ^##^ *p* ≤ 0.01). IL-1β, interleukin 1 beta; KC, keratinocytes-derived chemokine; IL6, interleukin 6; TNF-α, tumour necrosis factor; CCL2, monocyte chemoattractant protein-1 (MCP-1); iNOS, inducible nitric oxide synthase; Arg1, arginase 1.

Mice treated with cryo-MenSCs showed reduced inflammation in the DAD score (Supplementary Figures S4B, F), so different inflammatory mediators were analysed in lung tissue homogenates (Figure 6). Although not significant in some cases, overall, the profile of pro-inflammatory mediators suggests an immunomodulatory effect after treatment with cryo-MenSCs. On day 2, a trend of increased levels of mediators in lung tissue (pg/mg total protein±SEM) such as IL-1β (537 ± 230), KC (466 ± 150), IL-6 (63 ± 14), TNF-α (298 ± 19), and CCL2 (361 ± 32) was observed in the RL group compared with the CTRL group; interestingly, all these mediators showed a marked tendency to decrease in the animals treated with 1×10^5^ MenSCs/animal: IL-1β (86 ± 6), KC (168 ± 11), IL-6 (44 ± 5), TNF-α (290 ± 18), and CCL2 (283 ± 6). On day 7, for the RL and treated groups, it was observed that most of the mediators returned to levels similar to those for the CTRL group (Figure 6A). Figure 6F shows that cryo-MenSCs treatment favoured a reduction in iNOS expression levels compared with those in the ARDS-RL group at day 2 and an increase in Arg1 levels at day 7 (Figure 6G). Both molecules are indicative of pro- and anti-inflammatory macrophages M1 and M2-like, respectively (Orecchioni et al., 2019).

### Cryo-MenSCs treatment promotes proangiogenic and anti-apoptotic effects in lung-injured animals

**FIGURE 7:**
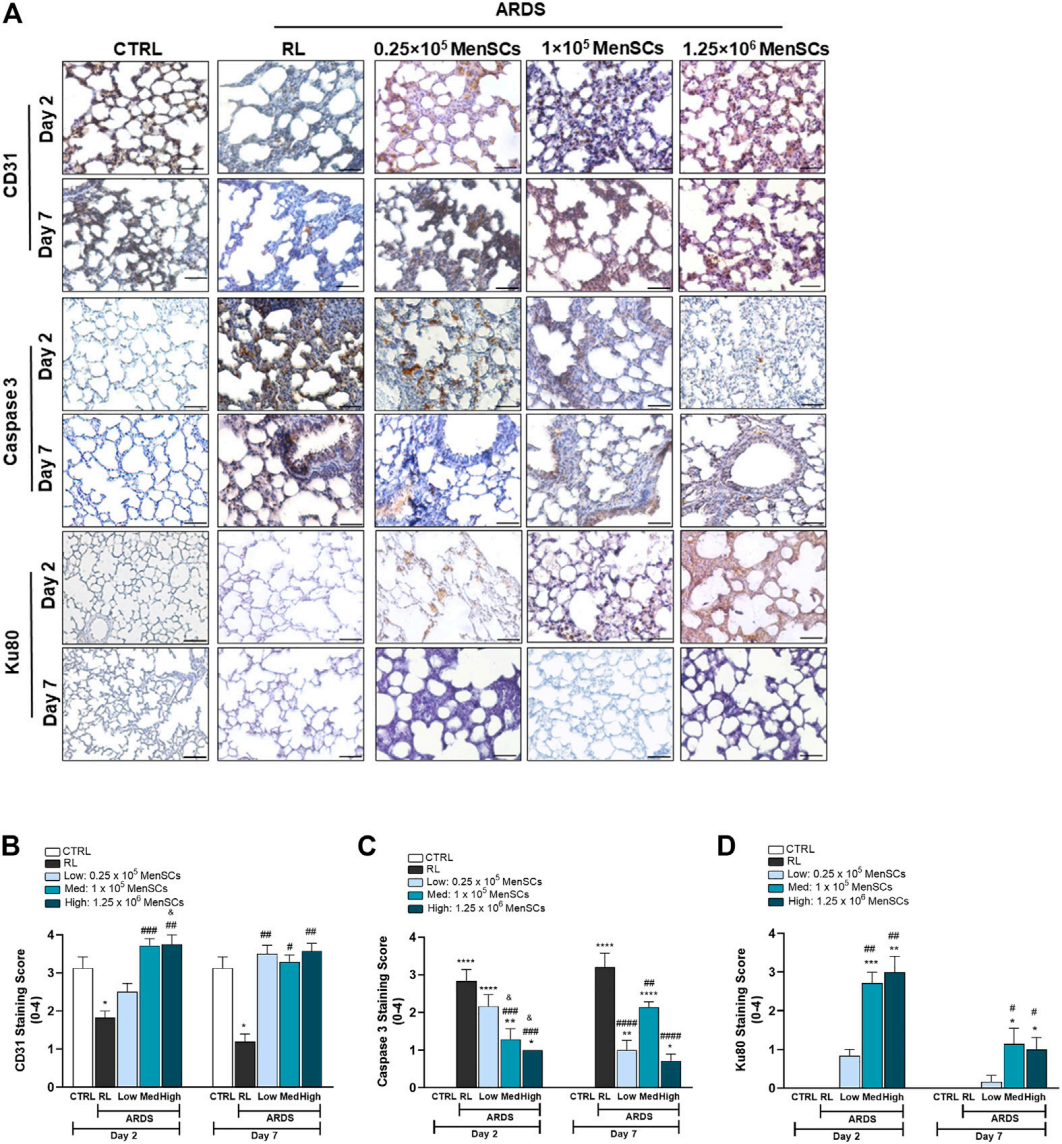
Pro-angiogenic and anti-apoptotic effect of cryopreserved MenSCs treatment and cryo-MenSCs persistence in lung-injured animals. The animals were euthanized on days 2 and 7 after LPS or RL injection, and the lungs were harvested and fixed for immunohistochemistry. **(A)** Representative images of lung staining with anti-CD31, anti-caspase-3 and anti-Ku80 antibodies for angiogenesis, apoptosis, and persistence evaluation, respectively. Scale bar, 100 μm. Semiquantitative scoring for **(B)** CD31, **(C)** caspase-3 based and **(D)** Ku80. Values are expressed as the mean ± standard error of the mean (n = 6–8, animals per group). ^*^Significantly different from the CTRL group (^*^ *p* ≤ 0.05, ^**^ *p* ≤ 0.01; ^****^ *p* ≤ 0.0001). ^**#**^Different from the RL group (^**#**^ *p* ≤ 0.05, ^##^ *p* ≤ 0.01, ^###^ *p* ≤ 0.001, ^####^ *p* ≤ 0.0001). ^&^Different from the low MenSCs dose (^&^ *p* ≤ 0.05).

In ARDS, the inflammatory environment mediates the disruption of epithelial cell junctions. It induces apoptosis of alveolar epithelial cells, increases production of reactive oxygen species, impairs lung barrier function, and increases vascular permeability (Matthay and Ware, 2015). Consequently, we evaluated cryo-MenSCs treatment on angiogenesis (anti-CD31) and apoptosis (anti-Casp-3) in lung tissue (Figures 7A–C). The data showed reduced CD31 (day 2 = 1.83 ± 0.17, *p* = 0.0451; day 7 = 1.2 ± 0.2, *p* = 0.0256) and increased Casp-3 (day 2 = 2.8 ± 0.31, *p* = 0.0001; day 7 = 3.2 ± 0.37, *p* = 0.0001) staining in the ARDS-RL group compared with the CTRL group at both endpoints of day 2 (CD31 = 3.13 ± 0.3; Casp-3 = 0.0) and 7 (CD31 = 3.13 ± 0.3; Casp-3 = 0.0). In contrast, cryo-MenSCs at all doses, except 0.25×10^5^ at day 2, increased angiogenesis compared with the untreated group (ARDS-RL) (Figure 7B). As Figure 7C shows, the cryo-MenSCs group at day 2 experienced a decrease in apoptosis at all doses studied, and this was most pronounced with doses of 1×10^5^ and 1.25×10^6^ cryo-MenSCs/animal. This decrease was preserved on day 7. Additionally, to complement the apoptosis and angiogenic analyses, we performed Western blot to detect PARP (nuclear poly (ADP-ribose) polymerase) and VEGF-A (vascular endothelial growth factor), respectively (Supplementary Figure S5).

### Persistence of cryo-MenSCs in lung tissue of treated animals

To examine the persistence of cryo-MenSCs after injection, lung tissue samples were stained with anti-human Ku80 antibody. As shown in Figure 7D, Ku80 was detected in all treated groups. On day 2, mild positivity was observed for the lowest dose (0.83 ± 0.17), and moderate staining for 1×10^5^ cryo-MenSCs/animal (2.71 ± 0.29), and the highest dose (3 ± 0.41). As expected, on day 7, positivity dropped to less than half for all doses studied. Specifically, a Ku80 staining score of 0.17 ± 0.17 was obtained for 0.25×10^5^ cryo-MenSCs/animal; the score was 1.14 ± 0.40 for 1×10^5^ cryo-MenSCs/animal and 1 ± 0.31 for dose 1.25×10^6^ cryo-MenSCs/animal, suggesting persistence of cryo-MenSCs paracrine activity beyond 7 days.

## Discussion

**FIGURE 8:**
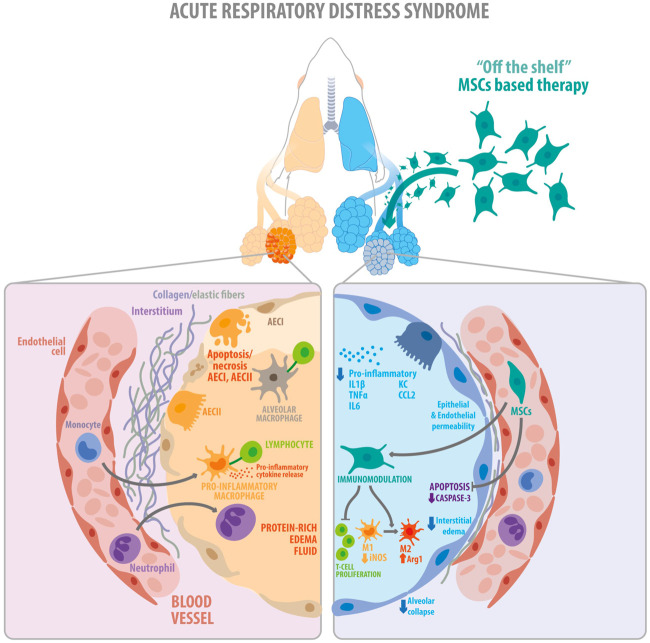
Schematic illustration showing the key findings of the study. The diagram summarizes the proposed therapeutic effects exerted by the clinical-grade cryo-MenSCs in a mouse model of ARDS. On the left, the figure shows the alveolar, interstitial, and capillary compartments of the lung, with protein-rich oedema fluid filling the injured alveolus in ARDS. On the right, cryo-MenSCs infused intravenously modulate tissue repair through immunomodulatory effects decreasing pro-inflammatory cytokines, suppression of T cell proliferation and macrophage polarization (M1→M2). Likewise, cryo-MenSCs produced a reduced remodeling associated with a diminution in collagen and elastic fibers and induced an increment in angiogenesis, reduction of apoptosis, alveolar collapse, and oedema (diminution of endothelium and epithelium permeability), all contribute to improving lung function impaired by ARDS. MSCs, mesenchymal stem cells; AEC (I, II), alveolar epithelial cells type I and type II, IL1β, interleukin 1 beta; TNFα, tumour necrosis factor alpha; IL6, interleukin 6; KC, keratinocytes-derived chemokine; CCL2, chemokine (C-C motif) ligand 2 (also referred to as monocyte chemoattractant protein 1); M1, pro-inflammatory macrophage type 1; M2, anti-inflammatory type 2.

The present study demonstrates that clinical-grade cryopreserved MenSCs retain their identity and purity and have similar biological properties as fresh cells *in vitro*. More importantly, in experimental ARDS, treatment with cryo-MenSCs was safe and improved survival and lung function, modulating inflammatory and remodeling processes, thereby increasing the translational value of our findings (Figure 8). Moreover, we identified that the minimal effective dose of cryo-MenSCs was 1×10^5^ cells/mouse or extrapolating by body weight, 4×10^6^ cells/Kg.

To date, there are still discrepancies in the literature regarding the impact of cryopreservation on the function of MSCs and, consequently, on their efficacy in experimental models and human clinical trials (François et al., 2012; Matthay et al., 2019). The maintenance or loss of cell potency after cryopreservation depends on the source of the cells, freezing and thawing methods, cryoprotectant, and reconditioning protocols of the final cellular product. These substantial variabilities could explain in part the discrepancies in the results observed in the published literature.

Our results showed that fresh MenSCs and cryo-MenSCs exhibited potential to undergo tri-differentiation and maintained the surface marker profiles as demonstrated for other adult MSCs (Goh et al., 2007). Determining the viability of cells after thawing is critical for clinical applications (Marquez-Curtis et al., 2015), for which at least 70% viability is generally accepted as the major release criterion (Robb et al., 2019). In a phase 2a trial, Matthay et al. (Matthay et al., 2019) found that 28-day mortality did not differ between patients with moderate to severe ARDS treated with or without MSCs. However, the authors note that the viability of the infused cryopreserved bone marrow MSCs ranged from 36–85%, which could partly explain the lack of overall efficacy observed in the treated group. On the other hand, a phase 1/2a trial study by Lanzoni *et al.* using frozen and thawed umbilical cord MSCs with viability >80% to treat patients with COVID-19 ARDS (mild to severe) reported that the treatment was associated with significantly improved patient survival and time to recovery (Lanzoni et al., 2021). The present work showed that fresh MenSCs and cryo-MenSCs display similar viabilities (>90%) and reduced apoptosis and senescence immediately after preparation. In agreement with other studies (Chinnadurai et al., 2014; Bahsoun et al., 2020), observed adhesion and clonogenic capacity were slightly diminished in cryo-MenSCs compared with fresh cells, and although a decrease in metabolic activity was observed in the short term, this effect was reversible and comparable with the fresh cells at the end of the experiment. Killer et al. (Killer et al., 2017) attributed this phenomenon to DMSO and demonstrated that metabolic activity increased in the days after thawing.

One of the mechanisms of action by which MSCs exert beneficial effects in ARDS lies in immune modulation (Laffey and Matthay, 2017), and MenSCs have been shown to be significant inhibitors of the inflammatory response (Luz-Crawford et al., 2016; Cuenca et al., 2018). Multiple factors, including growth factors, extracellular vesicles, chemokines, and apoptotic cells, contribute to the mechanisms of MSCs-mediated immunomodulation (Shi et al., 2018). Although we did not explore deeply into these mechanisms in this work, we observed *in vivo* a decreased inflammation at the histological level and a reduction in lung tissue inflammatory factors. This was also aligned with our *in-vitro* studies, where cryo-MenSCs remained suppressive of activated peripheral blood mononuclear cells (PBMCs) in co-culture assays similar to the fresh cells, as consistent with other authors (Cruz et al., 2015; Luetzkendorf et al., 2015; Gramlich et al., 2016; Chabot et al., 2017; Curley et al., 2017; Tan et al., 2019). Some data correlate the expression of CD146 with innately higher immunomodulatory capacity in BM-MSCs (Bowles et al., 2020). In the case of MenSCs, the expression of CD146 was similar in fresh and cryo-MenSCs (Supplementary Figure S6). However, there are still discrepancies about the effect of cryopreservation on the immunosuppressive properties of MSCs (François et al., 2012; Cruz et al., 2015; Luetzkendorf et al., 2015; Chinnadurai et al., 2016; Gramlich et al., 2016). It is relatively straightforward that cryopreserved and thawed cells have certain functional biological differences compared with those from continuous or fresh cultures; however, the real impact these discrepancies have at the therapeutic level is still under discussion and needs to be further explored.

To the best of our knowledge, no previous research has compared cryo-MenSCs with fresh MenSCs *in-vitro* and investigated the therapeutic effect of different doses of clinical-grade cryo-MenSCs in experimental ARDS. In contrast to previous studies in which MenSCs were administered a few hours after acute lung injury (Xiang et al., 2017; Ren et al., 2018), thus not considering the time course of lung damage, in the present study, cryo-MenSCs were injected 1 day after induction of ARDS when changes in lung mechanics, inflammation, and remodeling were already established and following during several days after cryo-MenSCs infusion. Cryo-MenSCs were infused IV because no differences have been reported between the IV and intratracheal routes of administration (Devaney et al., 2015). In addition to differing application modes, MSCs dose-response data show inconsistency among animal models of acute lung injury, ranging from 2×10^6^ to 5×10^9^ cells/Kg (Fengyun et al., 2020). We evaluated five doses of cryo-MenSCs, differing to previous studies that tested only one dose of 1×10^6^ cells/mouse (40×10^6^ MenSCs/Kg) (Xiang et al., 2017; Ren et al., 2018). Overall, the best correspondence between minimum dose and efficacy was observed at 1×10^5^ cryo-MenSCs/mouse (4×10^6^ cryo-MenSCs/Kg), ten times lower than previously reported (Xiang et al., 2017; Ren et al., 2018).

In the present study, cryo-MenSCs treatment showed attenuated histopathological alterations of the lung with improved lung mechanics parameters, which led to the recovery of lung function and enhanced survival. Also, cryo-MenSCs modulated different inflammatory mediators associated with ARDS. The diminished inflammation after cryo-MenSCs treatment was accompanied by decreased α-SMA, collagen, and elastic fiber content, reduced medial and adventitial layers of small pulmonary arteries, and an anti-apoptotic and pro-angiogenic effect in the lungs. These findings are consistent with previous studies that suggested that MenSCs administration decreased Casp-3 and IL-1β and could upregulate KGF expression to improve microvascular permeability. Likewise, serum biochemical parameters (Supplementary Figure S7) showed no adverse effects on metabolism or liver and kidney function, conferring good treatment tolerability with cryo-MenSCs without organ dysfunction initiation.

The highest dose studied, 1.25×10^6^ cryo-MenSCs/mouse (50×10^6^ cryo-MenSCs/Kg), did not lead to advantages over the 1×10^5^ cryo-MenSCs/mouse dose. Further study is needed to elucidate why this phenomenon occurs and highlights the importance of dose-response analysis for cell-based therapies. Although it is challenging to escalate from rodent studies to the dose required in humans, the dose of 4 million MenSCs/Kg would be feasible to produce and infuse in patients with ARDS.

Recently, Xu et al. (Xu et al., 2021), in an exploratory clinical trial, reported that treatment with 1×10^6^ MenSCs/Kg (90 × 10^6^ total) was safe and lowered the mortality of COVID-19 patients, reduced dyspnea in the short-term, improved chest imaging results during the 1-month study period but failed to promote significant changes in inflammatory factors (Xu et al., 2021). The preclinical safety and efficacy of animal studies supporting the use of this dose are not clarified; perhaps a different dose of MenSCs could have given a more substantial effect. This highlights the importance of MSCs-based therapies to identify the minimum dose with maximum efficacy and the scaling-up studies from small to human-relevant larger animal models.

## Conclusion

From a translational perspective, our results showed that clinical-grade cryopreserved MenSCs retain their biological properties and exert a therapeutic effect in mild to moderate experimental ARDS. The minimal effective dose of 4×10^6^ cells/Kg was shown to be well-tolerated, safe, and effective, favoring an improvement in lung function and survival, ameliorating inflammation, remodeling, and lung damage. Transforming cryopreserved MenSCs from an experimental product into a clinical treatment formulation would have great potential for improving patients with ARDS.

## Data Availability

The original contributions presented in the study are included in the article/Supplementary Material, further inquiries can be directed to the corresponding author.
