# Supplementary material for: Safety and efficacy of clinical-grade, cryopreserved menstrual blood mesenchymal stromal cells in experimental acute respiratory distress syndrome
Source: Front Cell Dev Biol. 2023 Jan 30;11:1031331. doi: 10.3389/fcell.2023.1031331 (PMC9923023; doi:10.3389/fcell.2023.1031331)
Supplement: Supplementary file 1 [file DataSheet1.pdf]

Supplementary material

I. Supplemental Experimental Procedures

Differentiation

All MSCs were evaluated for their capacity to differentiate into adipocytes, osteocytes, and chondrocytes using StemPro Differentiation Kits (Gibco, Waltham, MA, USA) under the manufacturer's instructions and as previously reported (Alcayaga-Miranda et al., 2015). After 14–21 days in culture, MSCs were stained for adipogenic, osteogenic, and chondrogenic differentiation using Oil Red O, Alizarin Red, and Safranin O staining (all from Sigma-Aldrich, St Louis, MO, USA), respectively. Quantification of color intensity of the digital images was calculated using the Image J Software (NIH).

Cell surface characterization

Immunophenotyping of MSCs was performed by flow cytometry using a FACSCanto II cytometer (BD Biosciences, Franklin Lakes, NJ, USA) after staining with specific antibodies CD105, CD90, CD73, CD44, HLA-DR, CD34, HLA- ABC, CD19, CD14, and CD45 (all from BD Pharmingen, San Diego, CA, USA) using standard protocols. Additionally, was added the anti-CD146 (BD Pharmingen, San Diego, CA, USA) for specific experiments. A LIVE/DEAD Fixable Dead Cell Stain Kit (Invitrogen, Carlsbad, CA, USA) was used to assess the viability of cells. The data acquired were analyzed using FlowJo software V10 (Tree Star, Ashland, OR, USA).

Cell proliferation assay

Cell proliferation assay was performed as previously described (Cuenca et al., 2018). Briefly, MSCs were cultured (1×10^3/well) in a 96-well plate (Falcon) in a final volume of 200 μL/well of DMEM supplemented with 10% fetal bovine serum (FBS), respectively. A Quick Cell

Proliferation Assay Kit (BioVision, Milpitas, CA, USA) was used to assess metabolic activity at different time points, following the manufacturer's instructions.

Adhesion ability

Cells were seeded in a six-well culture plate (Falcon) with supplemented alpha MEM and allowed to attach for 24 h at 37°C in a humidified atmosphere at 5% CO_2. At 24 h, non-adherent cells were removed by washing the wells with phosphate-buffered saline. The adherent cells were trypsinized and counted and the percentage of adherent cells used for the comparison assessment was calculated.

Colony-forming units assay

MSCs were evaluated for frequency of fibroblast colony-forming units (CFU-F) as described previously (Alcayaga-Miranda et al., 2015; González et al., 2015). CFU-F were evaluated in a serial dilution assay: 25–250 cells/well were seeded in a six-well plate (Falcon) and cultivated for 14 days. MSCs were fixed in 70% methanol and stained with 0.5% crystal violet (Sigma-Aldrich) in 10% methanol for 20 min. After five washes, colonies formed by more than 50 fibroblast-like cells were counted under a light microscope at low magnification.

Karyotyping

Cryopreserved and fresh cells were screened for chromosomal abnormality through karyotyping with a standard GTG banding protocol. Briefly, cryopreserved cells were thawed and grown for at least 24 h in a-MEM supplemented with 10% FBS to allow cells to proliferate to approximately 60%. The same confluence was used for fresh cells. The MenSCs undergoing active cell division were blocked at metaphase by adding Colcemid solution (10 µg/mL; Gibco Invitrogen) to each culture. After 12 h of incubation at 37°C, cells were detached before proceeding with the karyotyping protocol. Metaphases were karyotyped following the recommendations of the

International System for Human Cytogenetic Nomenclature (ISCN 2020) (McGowan-Jordan et al., 2020).

T cell proliferation assay

The immunosuppressive capacity of MSCs was assessed in a T cell proliferation assay as reported previously (Cuenca et al., 2018). MSCs, pre-stimulated with 10 ng/mL IL-1β and TNF-α (PeproTech, East Windsor, NJ, USA) (control: no stimulation), were seeded in defined cell numbers and left to adhere. Peripheral blood mononuclear cells (PBMCs) were isolated from heparinized human peripheral blood samples (healthy donors) by density gradient centrifugation (Ficoll; GE Healthcare, Amersham, UK). PBMCs were stained with Cell Trace Violet (Molecular Probes, Springfield, MA, USA) according to the manufacturer's instructions and co-cultured with MSCs (MSC/T cell ratios, 1:5 and 1:20) in supplemented RPMI 1640 medium (10% FBS, 1% L-glutamine, 1% penicillin/streptomycin, 1% nonessential amino acids, 100 mM sodium pyruvate, 25 mM β-mercaptoethanol) (all from Gibco). The proliferation of T cells was stimulated with phytohemagglutinin (PHA; 15 µg/mL, Sigma-Aldrich). After 3–4 days, cells were harvested and analysed by flow cytometry. The percentage of CD45+CD3+ proliferative T cells was determined using FlowJo software V10 (Tree Star).

Apoptosis assay

Briefly, fresh and cryo-MenSCs (1×10^5 cells) were stained with 0.5 µL of Annexin V APC (BioLegend, San Diego, CA, USA) and 7-aminoactinomycin D (7AAD) viability staining solution (BioLegend) at 1 µg/mL in 100 µL of Annexin V binding buffer (BioLegend) by incubating for 20 min at room temperature. Then, 100 µL of Annexin V binding buffer was added, and the cell suspension was analysed on a BD FACS Canto II (Becton Dickinson, San Diego, CA, USA) flow cytometer. Data analysis was performed using the FlowJo software version 10 (Tree Star).

Senescence assay

Cell senescence was performed as described previously (Bartolucci et al., 2017). A histochemical staining kit (Sigma), with 10,000 cells per well seeded on 24-well plates, was used to complete the assay using senescent cells. After 5 h, the staining mixture was added for detection of SA-β-galactosidase and incubated overnight at 37°C following the manufacturer’s instructions. Positively stained cells were counted, and the percentage of cells expressing SA-β-galactosidase (senescent cells) in the total cell number of each sample was calculated.

Western blot analyses

PARP (nuclear poly (ADP-ribose) polymerase) and VEGF (vascular endothelial growth factor) protein expressions were determined by Western blot in lung tissue homogenate. Briefly, lung tissue samples were homogenized in RIPA buffer containing 1% v/v of protease and phosphatase inhibitor cocktail (Thermo Fisher Scientific, Waltham, MA, United States, Cat. # 78440). Total protein concentrations were determined with Pierce BCA Protein Assay Kit (Thermo Scientific, Rockford, IL, United States, Cat. #23225), and 20 μg of each lysate was mixed with Laemmli buffer 5×, heated for 5 min at 95◦C, separated on 4–20% gels by SDS-PAGE, and transferred to PVDF membranes (GE Healthcare Limited, Chicago, IL, United States, Cat. #RPN303F).

Primary antibodies used were PARP (1:1000; Cell Signaling Technology, Danvers, MS, United States. Cat. #9542), VEGF (1:1000; Clone #5C3.F8, Abcam Inc., Cambridge, United Kingdom. Cat. # ab3109), and glyceralde- hyde-3-phosphate dehydrogenase (GAPDH) (1:1000; Clone #D16H11, Cell Signaling Technology, Danvers, MS, United States. Cat. #5174) to confirm equal loading of the gels. For fluorescence detection of proteins, InvitrogenTM Goat anti-Rabbit (H + L) Highly Cross-Adsorbed secondary antibody and Alexa Fluor Plus 800 was used (1:25,000;

Thermo Fisher Scientific, Waltham, MA, United States, Cat. #A32735). Protein signals were captured using a LI-COR Odyssey imaging system (LI-COR Biosciences, Lincoln, NE, United States) and quantified by densitometric analysis with the ImageJ software (NIH).

Histology

The animals were euthanized on days 2 and 7 after lipopolysaccharide (LPS) injection. The lungs were harvested and fixed for Van Gieson stain and immunohistochemistry of alpha-smooth muscle actin (α-SMA). Van gieson was used to determine the wall thickness for the small pulmonary arteries (150–250 μm of internal diameter) calculated as previously described (Castillo-Galán et al., 2020). Briefly, the percentage of vascular smooth muscle was calculated as follows: Medial layer (%) = [(external muscle area − internal area)/external muscle area] × 100; Adventitial layer (%) = [(external area – external muscle area)/external area] × 100, where the external area, external muscle area, and the internal area are the external whole artery area, external and internal boundaries of the tunica media, respectively. Images were captured at 20× with a microscope Olympus BX-41 coupled (Olympus, Shinjuku-ku, Tokyo, Japan) to a digital camera (Jenoptik Progres C3). Ten to fifteen representative small pulmonary arteries from each animal were selected for these analyses. The analysis of the microphotographs was performed with the software Image Pro-Plus 6.2 (Media Cybernetics, Inc., Rockville, MD, USA).

Immunohistochemistry was performed to detect α-SMA (EPR5368 rabbit polyclonal 1:400; Abcam, Cambridge, MA, USA) using the Dako Envision system (Dako,Carpinteria, CA, USA) according to the manufacturer’s recommendations. Immunoreactive sections were visualized with diaminobenzidine (DAB) solution (Dako) and counterstained with hematoxylin. Using a microscope (Olympus BX41) coupled with a digital camera, 5-6 pulmonary tissue images per animal were digitally acquired at 40× using the NIS-Elements software (Nikon, Minato, Tokio,

Japan). The α-SMA immunoreactivity in the pulmonary epithelial airway was determined using the ImageJ software (NIH).

References

Alcayaga-Miranda, F., Cuenca, J., Luz-Crawford, P., and et al. (2015). Characterization of menstrual stem cells: Angiogenic effect, migration and hematopoietic stem cell support in comparison with bone marrow mesenchymal stem cells. *Stem Cell Res. Ther.* 6, 32. doi:10.1186/s13287-015-0013-5.

Bartolucci, J., Verdugo, F. J., González, P. L., Larrea, R. E., Abarzua, E., Goset, C., et al. (2017). in Patients With Heart FailureNovelty and SignificanceSafety and Efficacy of the Intravenous Infusion of Umbilical Cord Mesenchymal Stem Cells. *Circ. Res.* 121, 1192–1204. doi:10.1161/CIRCRESAHA.117.310712.

Castillo-Galán, S., Arenas, G. A., Reyes, R. V, Krause, B. J., and Iturriaga, R. (2020). Stim-activated TRPC-ORAI channels in pulmonary hypertension induced by chronic intermittent hypoxia. *Pulm. Circ.* 10, 13–22. doi:10.1177/2045894020941484.

Cuenca, J., Le-Gatt, A., and Castillo, V, et al. (2018). The reparative abilities of menstrual stem cells modulate the wound matrix signals and improve cutaneous regeneration. *Front. Physiol.* 9, 464. doi:10.3389/fphys.2018.00464.

González, P. L., Carvajal, C., Cuenca, J., Alcayaga-Miranda, F., Figueroa, F. E., Bartolucci, J., et al. (2015). Chorion Mesenchymal Stem Cells Show Superior Differentiation, Immunosuppressive, and Angiogenic Potentials in Comparison With Haploidentical Maternal Placental Cells. *Stem Cells Transl. Med.* 4, 1109–1121. doi:10.5966/sctm.2015-0022.

McGowan-Jordan, J., Hastings, R. J., and Moore, S. (2020). *An International System for Human Cytogenomic Nomenclature (2020).* , ed. S. M. Jean McGowan-Jordan, Ros J.Hastings S.Karger.

II. Supplementary Figures

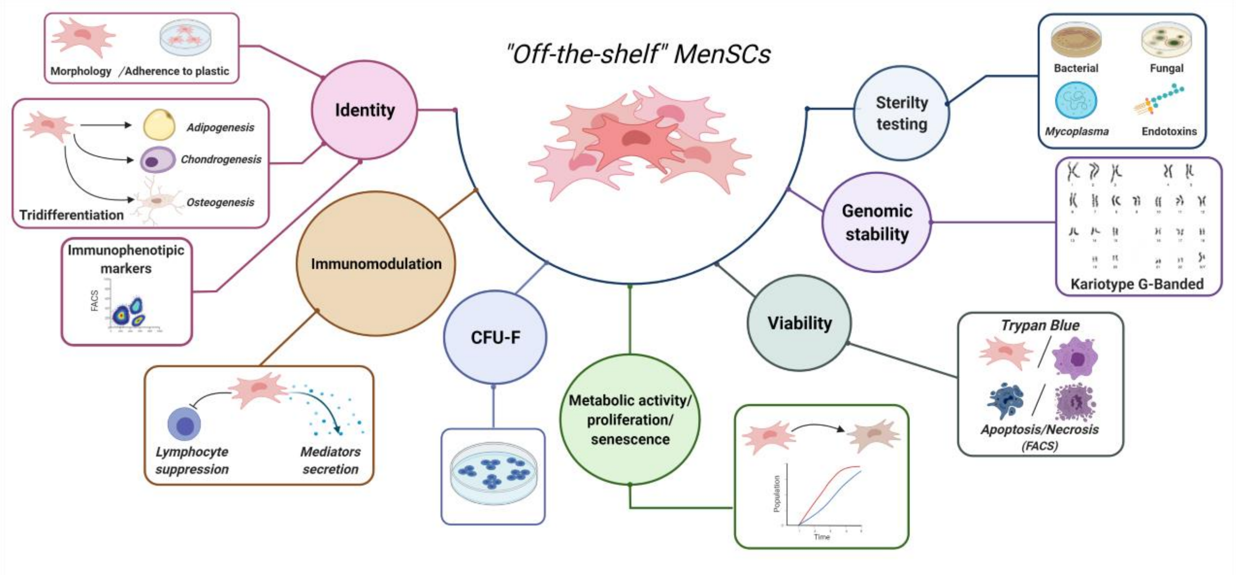

Supplementary Figure S1. Characterization *in vitro* of clinical-grade mesenchymal stem cells derived from menstrual blood (MenSCs). Evaluation of identity, viability, purity, potency (immunomodulation), metabolic/proliferative capacity, and genomic stability was performed in cryopreserved and fresh MenSCs. The good manufacturing practice guidelines of the laboratory indicate that MSCs should be monitored during the *in vitro* expansion process to analyze for any product contamination. Hence, sterility quality testing was carried out to ensure that MSC-based products were not infected after manufacturing. CFU-F, colony-forming unit-fibroblast. Figure created with BioRender.com.

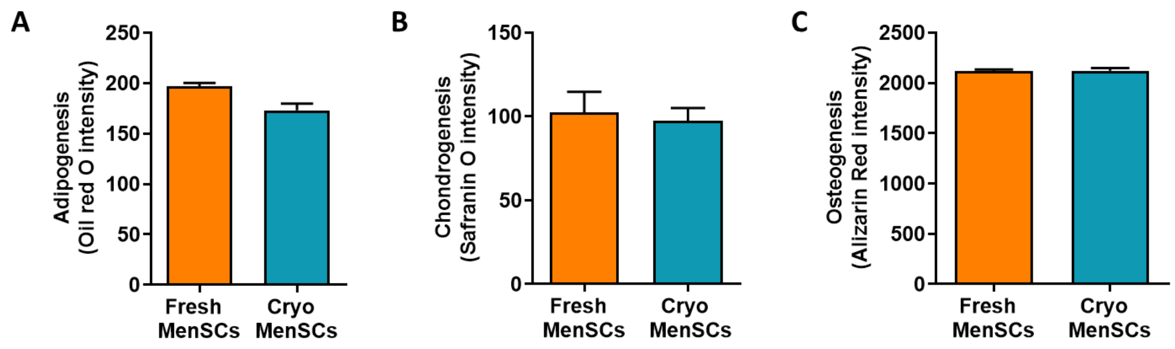

Supplementary Figure S2. Quantification analysis of MenSCs tri-lineage differentiation. *In vitro* differentiation potential of Fresh and Cryopreserved MenSCs towards osteogenic, adipogenic, and chondrogenic lineages. (a) Adipogenesis; (b) Chondrogenesis, and (c) Osteogenesis. Quantification of color intensity of the digital images was calculated using the Image J Software. Values are expressed as mean ± SEM (3 different MenSCs donors).

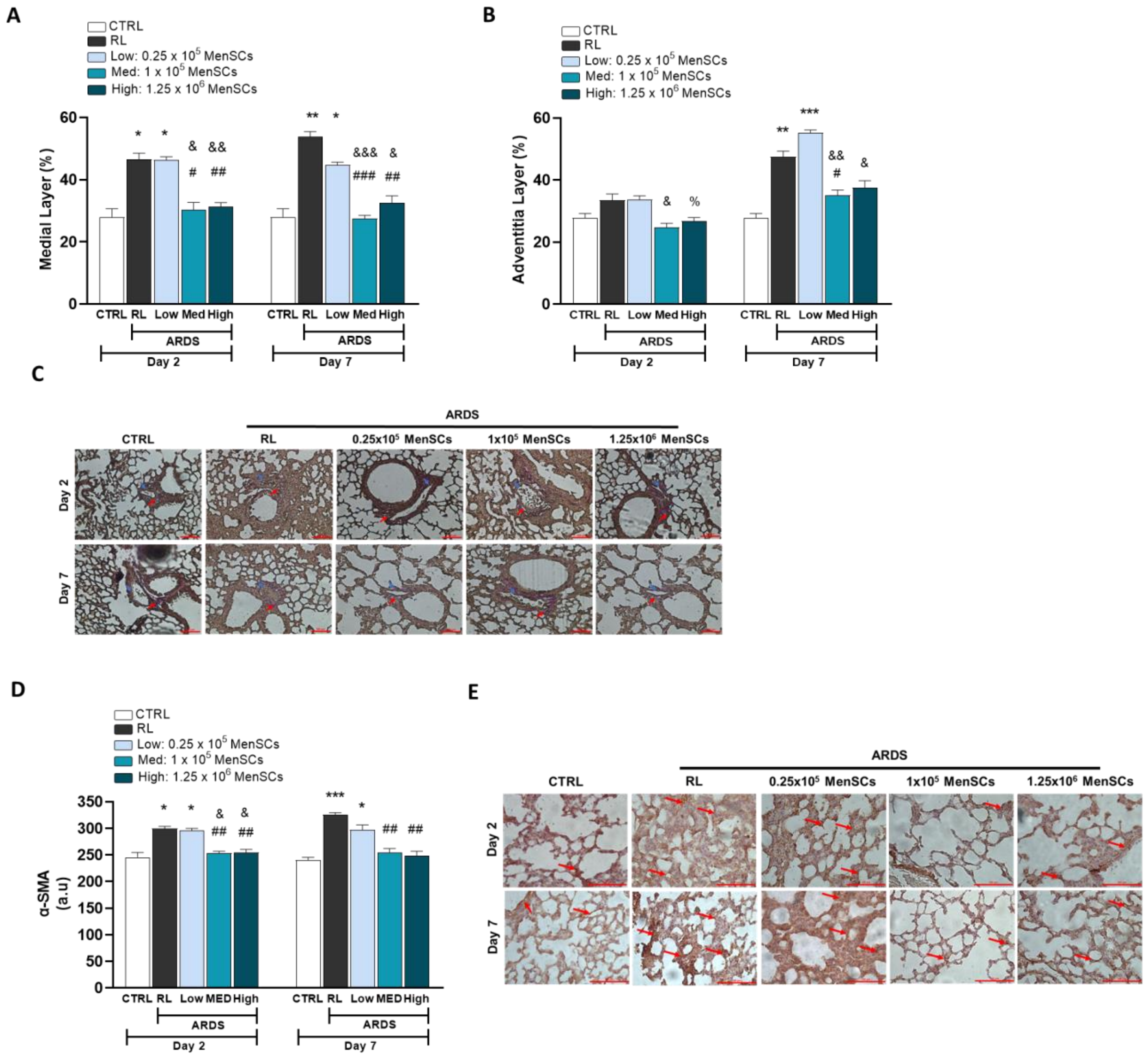

Supplementary Figure S3. Reduction in remodeling after cryo-MenSCs treatment. The animals were euthanized on days 2 and 7 after lipopolysaccharide (LPS) injection. The lungs were harvested and fixed for Van Gieson stain and immunohistochemistry of alpha-smooth muscle actin (α-SMA). Determination of the percentage of (a) medial and (b) adventitial layer of small pulmonary arteries. (c) Representative images of lung Van Gieson staining. Scale bar, 100 μm. The blue and red arrows indicate the medial and adventitial layers, respectively. (d) Quantification of α-SMA (red arrows) in the different study groups (a.u, arbitrary units). (e) Representative images

of α-SMA immunoreactivity in pulmonary epithelial airways. Scale bar, 100 μm. Values are expressed as the mean ± SEM (n=4 animals/group). *Significantly different from the CTRL group (*$p\leq0.05$,**$p\leq0.01$, ***$p\leq0.001$); #Different from the RL group (#$p\leq0.05$, ##$p\leq0.01$, ###$p\leq0.001$); &Different from the Low group (&$p\leq0.05$, &&$p\leq0.01$; &&&$p\leq0.001$; %Different from the Medium group (%$p\leq0.05$).

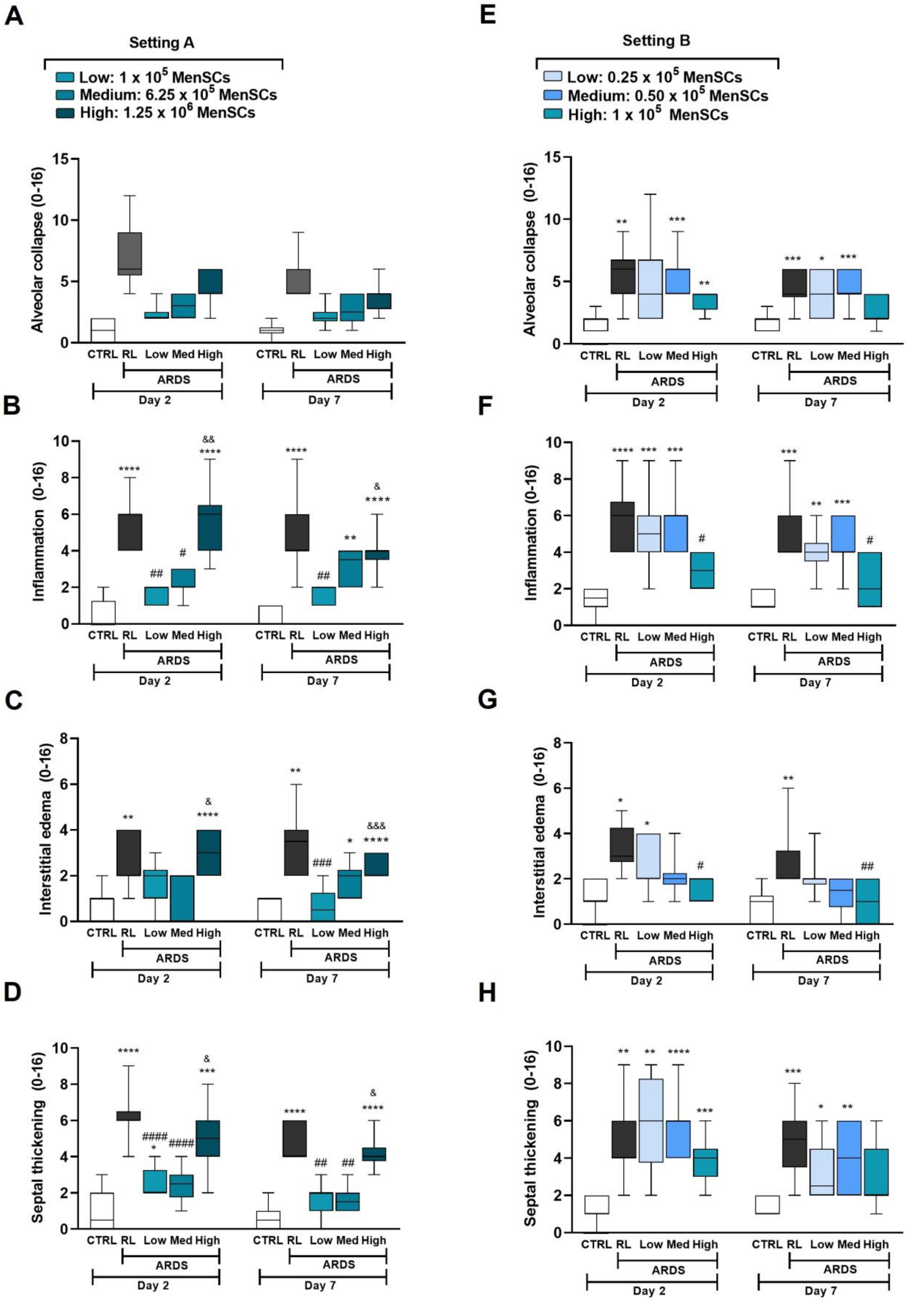
A
Setting A
Low: 1 x 10^5 MenSCs
Medium: 6.25 x 10^5 MenSCs
High: 1.25 x 10^6 MenSCs
Alveolar collapse (0-16)
CTRL RL Low Med High
ARDS
Day 2
Day 7
B
Inflammation (0-16)
C
Interstitial edema (0-16)
D
Septal thickening (0-16)
E
Setting B
Low: 0.25 x 10^5 MenSCs
Medium: 0.50 x 10^5 MenSCs
High: 1 x 10^5 MenSCs
F
G
H

Supplementary Figure S4. Treatment with cryopreserved menstrual blood mesenchymal stromal cells (cryo-MenSCs) reduces the histological parameters of severity in lung-injured animals. The score for each parameter at both settings, A and B: (a,e) alveolar collapse, (b,f) inflammatory infiltration, (c,g) interstitial oedema, and (d,h) septal thickening, was calculated as the product of severity and extent of each feature and ranged from 0 to 16. Values are expressed as medians, interquartile ranges (n=10 animals/group). *Significantly different from the CTRL group (*p≤0.05, **p≤0.01, ***p≤0.001; ****p≤0.0001). #Different from the RL group (#p≤0.05, ##p≤0.01, ###p≤0.001, ####p≤0.0001). &Different from the low MenSCs dose (&p≤0.05, &&p≤0.01, &&&p≤0.001).

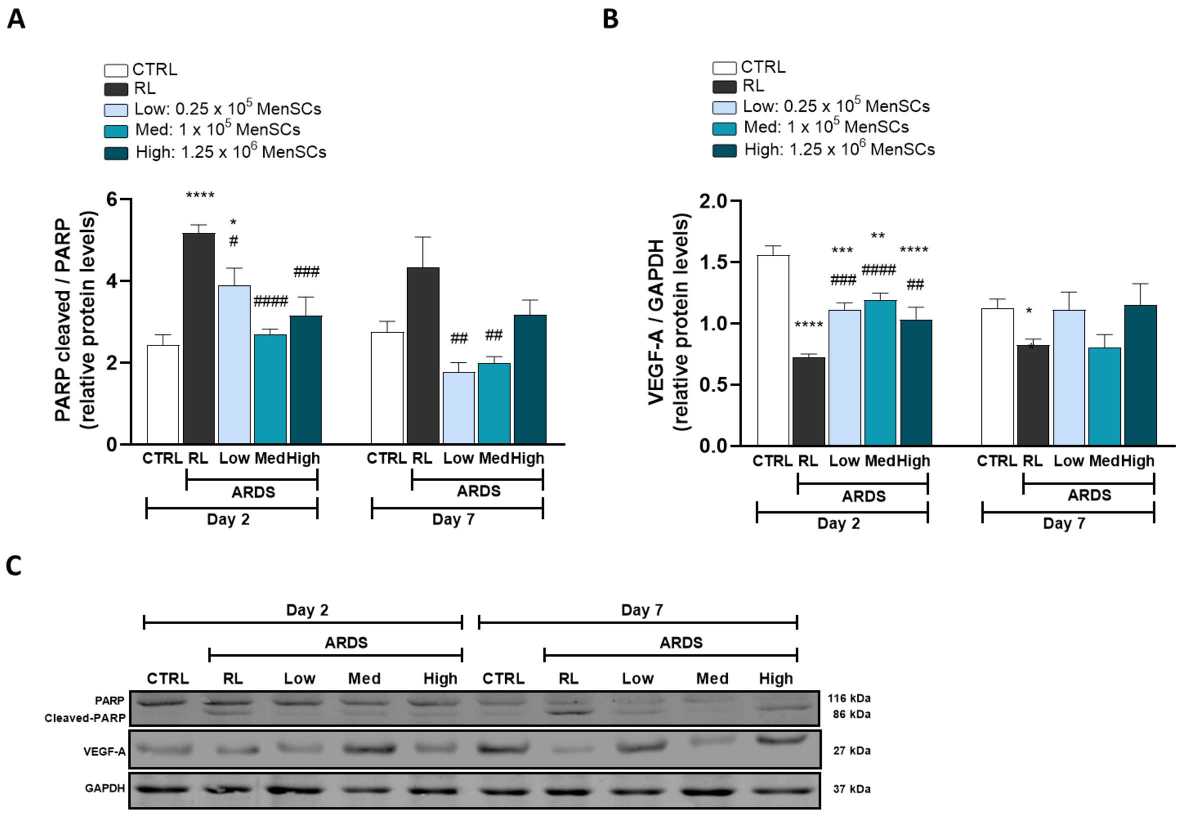

Supplementary Figure S5. Western blotting results of PARP and VEGF-A. Densitometry graph showing the relative protein levels of (a) PARP and (b) VEGF-A. (c) Representative

immunoblot. Values are expressed as mean ± SEM (n=6-9 animals/group). *Significantly different from the CTRL group (*p≤0.05, **p≤0.01, ***p≤0.001; ****p≤0.0001). #Different from the RL group (#p≤0.05, ##p≤0.01, ###p≤0.001, ####p≤0.0001). PARP, nuclear poly (ADP-ribose) polymerase; VEFG, vascular endothelial growth factor.

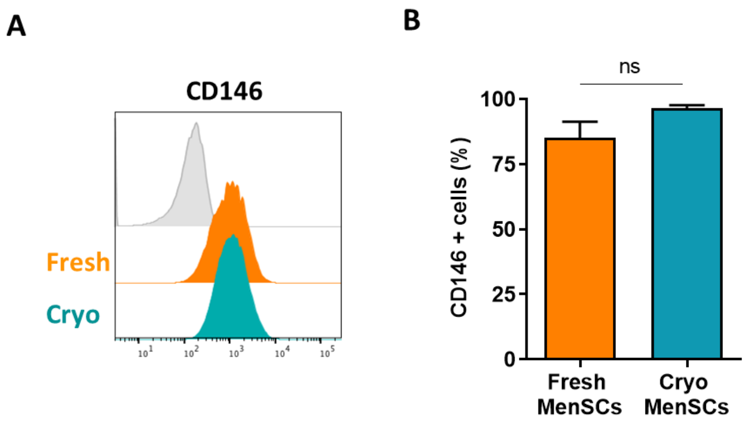

Supplementary Figure S6. Determination of CD146 surface marker in MenSCs. The analysis of the cell surface marker CD146 was performed in fresh and cryopreserved MenSCs through flow cytometry. (a) Representative histogram of the CD146 marker in Fresh MenSCs (orange) and Cryo-MenSCs (blue); autofluorescence control (grey) is also shown. (b) Percentage of CD146 positive cells of 3 different MenSCs donors. Values are expressed as mean ± SEM. ns: not significant.

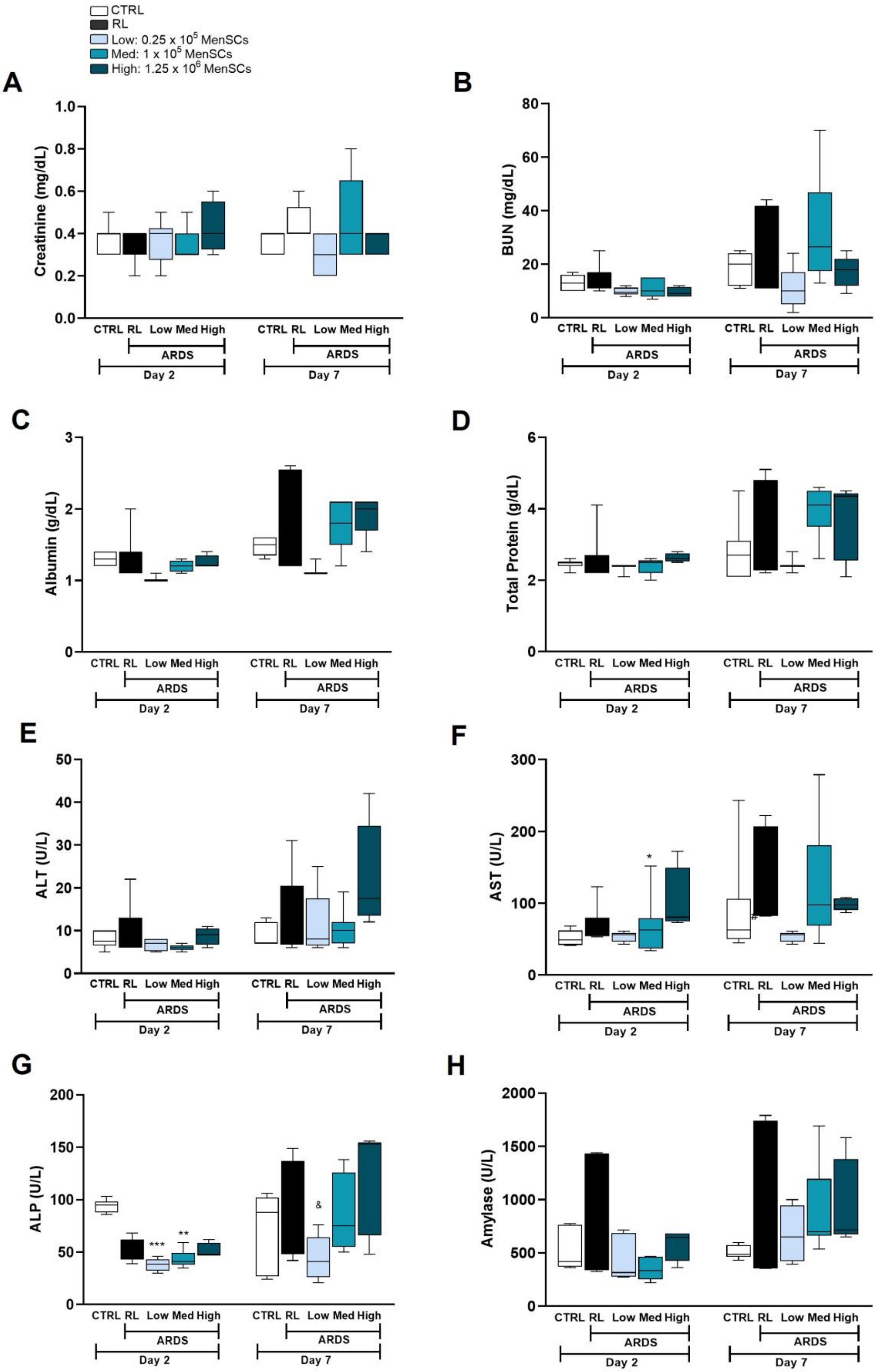

CTRL
RL
Low: 0.25 x 10^5 MenSCs
Med: 1 x 10^5 MenSCs
High: 1.25 x 10^6 MenSCs
A
Creatinine (mg/dL)
B
BUN (mg/dL)
C
Albumin (g/dL)
D
Total Protein (g/dL)
E
ALT (U/L)
F
AST (U/L)
G
ALP (U/L)
H
Amylase (U/L)
CTRL RL Low Med High
ARDS
Day 2
Day 7

Supplementary Figure S7. Serum biochemical parameters after treatment with cryopreserved menstrual blood mesenchymal stromal cells (cryo-MenSCs) in lung-injured animals. The animals were euthanized on days 2 and 7 after lipopolysaccharide or Ringer's lactate injection, and serum was harvested from blood. The concentration in serum was determined of (a) creatinine, (b) blood urea nitrogen (BUN), (c) albumin, (d) total protein, (e) alanine aminotransferase (ALT), (f) aspartate aminotransferase (*AST*), (g) aspartate aminotransferase (ALP), (h) amylase. Values are expressed as medians, interquartile ranges (n=6–8, animals per group). *Significantly different from the CTRL group (*p≤0.05, **p≤0.01, ***p≤0.001). #Different from the RL group (#p≤0.05). &Different from the low MenSCs dose (&p≤0.05).
